# Modeling Strong Light-Matter Coupling in Correlated Systems: State-Averaged Cavity Quantum Electrodynamics Complete Active Space Self-Consistent Field Theory

**DOI:** 10.1021/acs.jctc.5c00927

**Published:** 2025-08-30

**Authors:** Nam Vu, Kenny Ampoh, Mikuláš Matoušek, Libor Veis, Niranjan Govind, Jonathan J. Foley

**Affiliations:** † Department of Chemistry, 14727 University of North Carolina Charlotte, Charlotte, North Carolina 28223, United States; ‡ J. Heyrovský Institute of Physical Chemistry, 86875 Academy of Sciences of the Czech Republic, v.v.i., Dolejškova 3, 18223 Prague 8, Czech Republic; ¶ Faculty of Mathematics and Physics, Charles University, 12116 Prague 2, Czech Republic; § Physical and Computational Sciences Directorate, 6865 Pacific Northwest National Laboratory, Richland, Washington 99352, United States; ∥ Department of Chemistry, 14727 University of North Carolina Charlotte, Charlotte, North Carolina 28223, United States

## Abstract

The description of strongly correlated systems interacting with quantized cavity modes poses significant theoretical challenges due to the combinatorial scaling of the electronic and photonic degrees of freedom. Recent advances addressing this complexity include cavity quantum electrodynamics (QED) generalizations of complete active space configuration interaction and density matrix renormalization group methods. In this work, we introduce a QED extension of state-averaged complete active space self-consistent field theory, which incorporates cavity-induced correlations through a second-order orbital optimization framework with robust convergence properties. The method is implemented using both photon number state and coherent state representations, with the latter showing robust origin invariance in the energies, regardless of the completeness of the photonic Fock space. The implementation enables symmetry-free orbital relaxations to account for photon-mediated symmetry breaking in polaritonic systems. Numerical validation on lithium hydride, hydroxide anion, and magnesium hydride cation demonstrates that this method achieves significantly improved accuracy in modeling ground-state and polariton potential energy surfaces compared with QED-CASCI in a fixed orbital basis. In these studies, we reach sub kcal/mol accuracy in potential energy surface in much smaller active spaces than are required for QED-CASCI. This advancement provides a more robust approach for studying cavity-altered chemical landscapes for ground and excited strongly coupled systems.

## Graphical abstract

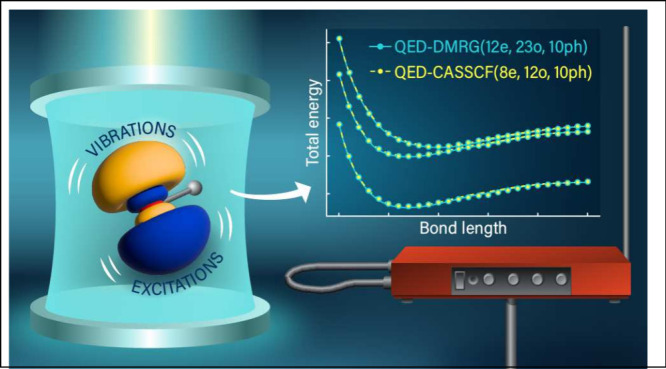

## Introduction

Strong coupling between molecules and quantized cavity-confined photon modes leads to incredibly rich phenomenology with promising applications for chemistry and quantum information science.^1^ Examples of this phenomenology include modification of (ground-state and excited/polariton) chemical reactivity,^2–8^ altered energy transport properties,^9–16^ and cavity-mediated intermolecular entanglement.^17,18^ Progress in understanding the properties and potential applications of molecules under strong light-matter coupling requires coordinated theoretical and experimental efforts to understand and exploit these complex interactions.^19–25^

Broadly speaking, there are two complementary domains for achieving strong light-matter coupling in molecular systems, depending on the volume of the cavity and the number of molecules that are coupled to it. In the few molecule regime, one or a small number of molecules are coupled to cavities with mode volumes *V* on the scale of 10^–1^–10^2^ nm^3^. Landmark experiments in this domain have been achieved with plasmonic picocavities^26,27^ and scanning tunneling microscope break-junctions.^28^ In such cases, the per-molecule interaction strength, quantified by λ = $\sqrt{1/\epsilon_{0}V}$ is on the order of 10^–3^–10^–1^ atomic units and the electric field uncertainty of the cavity vacuum state, $\sigma_{\mathrm{E⃗}}=\sqrt{\frac{\hbar \omega }{2\epsilon_{0}V}}$ , is on the order of 10^10^ V/m for modes with optical frequencies. Electronic coupling to such cavity modes is strong enough to modify the internal molecular structure^8,29,30^ and intermolecular forces,^31^ necessitating a sophisticated ab initio treatment of the molecular structure coupled to the cavity fields, the so-called “ab initio cavity quantum electrodynamics (aiQED)” approaches that include the method we introduce in this paper. The collective regime, which is also more experimentally accessible, involves the collective coupling of a very large number of molecules (≫10^6^) to one or more common cavity modes. Because the mode volumes for collective strong coupling are necessarily much larger, the per-molecule coupling (quantified again by λ) is quite small. A number of theoretical challenges distinct from aiQED methods arise in this domain as well.^19,20,32–38^ While it is not always clear under which circumstances cavity-induced/polaritonic effects play a nontrivial role in the collective coupling regime,^39^ theoretical studies have identified conditions under which collective strong coupling conditions can lead to strongly localized cavity effects.^40,41^ These predictions are consistent with the recent experimental observation that vibrational strong coupling in the collective limit can modify local intermolecular (van der Waals) interactions.^42^ In light of these findings, it is conceivable that aiQED approaches, if scaled to representative molecular ensembles, could help to elucidate some of these complex effects in the collective regime.

Recent methodological advances to aiQED approaches include quantum electrodynamics generalizations of Hartree–Fock (HF) theory,^29,43–45^ density functional theory (QEDFT^22,46–51^ and QED-DFT^52–55^ ), real-time^46,47,56–59^ and linear-response^52,60,61^ formulations of QED-TDDFT, configuration interaction (QED-CIS),^62^ real-time QED-TDCI,^63,64^ cavity QED extension of second-order Møller-Plesset perturbation theory and the algebraic diagrammatic construction,^65–67^ coupled cluster (QED-CC)^29–31,54,68–71^ and real-time^72^ and linear-response extensions of the QED-CC formalism,^73^ variational QED-2-RDM methods,^74^ and diffusion Monte Carlo,^75^ complete active space configuration interaction (QED-CASCI),^76^ density matrix renormalization group (QED-DMRG).^77^ There is also a complementary parametrized approach (pQED) that can utilize energies and dipole matrix elements from existing cavity-free quantum chemistry methods to project the Pauli–Fierz Hamiltonian onto a tensor product basis of many-electron states and photonic states.^20,78,79^ These important developments have helped the field to explore new and exciting potential avenues to control molecular structure and dynamics, which is responsible for fueling the momentum behind these development efforts. Recently, Ronca and co-workers introduced a state-specific QED generalization of the complete active space self-consistent field approach (SS-QED-CASSCF).^80^ In this work, we present a state-averaged QED generalization of the complete active space self-consistent field (SA-QED-CASSCF) theory. These two approaches can be seen as complementary: a state-specific QED-CASSCF formulation will variationally optimize a CAS wave function and orbital basis for a selected coupled electronic-photonic state, and a state-averaged QED-CASSCF formulation will variationally optimize these for some user-defined average over several coupled electronic-photonic states. We posit that our SA-QED-CASSCF approach can provide a robust tool for converging multiple strongly coupled states, such as polariton states with near degeneracies, and can provide a common orbital basis for these multiple states of interest to facilitate further computation of properties.

Our SA-QED-CASSCF approach builds upon the Werner–Meyer–Knowles (WMK) methodology.^81–84^ Our implementation entails two modifications to this established framework. First, we redefine the orbital gradient and Hessian to account for cavity-mediated interactions between photon modes and molecular orbitals. Second, we implement a specialized trust-region subproblem solver that properly handles “hard case” scenarios,^85^ which emerge when finding the global minimum of the trust-region subproblem becomes challenging due to near-singular Hessians. This issue is not uncommon in practice. For instance, molecules with high symmetry are often treated using lower point group symmetry, which can introduce rotations between orbitals of different irreducible representations of the higher point group and contribute negligibly to the optimization. In our study, cavity coupling can induce symmetry breaking, making near-singular Hessians a more frequent occurrence. The quantum chemistry literature addresses near-singular Hessian problems through various approaches, including level shifting, regularization techniques, and heuristic eigenvector selection procedures that prioritize numerical stability and step-size control over strict adherence to the trust-region subproblem solution.^86,87^ In contrast, our method rigorously finds the global minimum of the trust-region subproblem even in the “hard case”, ensuring that the second-order energy approximation always decreases while maintaining the theoretical convergence guarantees of the trust-region framework. This approach retains the WMK method’s favorable convergence properties while adapting to the unique requirements of polaritonic systems. The resulting QED-CASSCF framework enables systematic studies of cavity-mediated chemical phenomena, while balancing computational tractability with physical accuracy. Implementation details and challenges are outlined in the Theory section and discussed in greater detail in the Supporting Information, with particular emphasis on maintaining numerical stability when combining photonic and electronic degrees of freedom in symmetry-free orbital optimizations.

## Theory

The state-averaged QED-CASSCF approach is designed to approximate the energy eigenstates of the Pauli–Fierz Hamiltonian in the length gauge and within the dipole approximation, which results from transforming the minimal coupling Hamiltonian in the Coulomb gauge through the Power–Zienau–Woolley transformation^88–91^ under the long-wavelength approximation. This representation has been favored for aiQED approaches that are implemented with localized Gaussian basis sets,^20,24^ because the light-matter coupling arises through the molecular dipole operator, where the dipole operator itself has eigenstates that are localized in space. The Coulomb gauge representation of the Hamiltonian, by contrast, contains light-matter coupling through the molecular momentum operator, which has delocalized eigenstates that are less convenient to represent with localized Gaussian orbitals.^20^ The Coulomb gauge representation has been implemented in the context of QEDFT^47,92^ using real-space grids, but, to our knowledge, real-space implementations of many-body approaches for aiQED have not been developed. Recent advances in many-body methods for molecular electronic structure, including CASSCF, have employed real-space orbital representations to address limitations of Gaussian orbitals,^93^ suggesting a potential pathway toward real-space formulations of many-body aiQED approaches that would be compatible with both Coulomb and length gauges.

The length gauge Pauli–Fierz Hamiltonian captures the interaction between the molecular electronic Hamiltonian and one or more quantized radiation modes,

$${Ĥ}_{\mathrm{PF}}={Ĥ}_{e}+\overset{N_{\mathrm{modes}}}{\underset{P}{\sum }}\left(\omega_{P}{\mathrm{b̂}}_{P}^{†}{\mathrm{b̂}}_{P}-\sqrt{\frac{\omega_{P}}{2}}{\mathrm{d̂}}_{P}\left({\mathrm{b̂}}_{P}^{†}+{\mathrm{b̂}}_{P}\right)+\frac{1}{2}{\mathrm{d̂}}_{P}^{2}\right)$$ 1

In eq 1, *Ĥ*_e_ is the standard electronic Hamiltonian within the Born–Oppenheimer approximation, ω_*P*_ *b̂*_*P*_^†^ *b̂̂*_*P*_ is the bare Hamiltonian for the *P*th photon mode, where ω_*P*_ represents the frequency and *b̂*_*P*_^†^ and *b̂̂*_*P*_ are the raising and lowering operators for mode *P*. The final two terms, the bilinear coupling and the dipole self-energy (DSE), capture interactions between the photonic and the electronic degrees of freedom. In these terms, *d̂*_*P*_ = **λ**_*P*_ · **μ̂** couples the field associated with a given mode to the molecular dipole operator.^24,76,94^ We restrict our attention to the single-mode case for the subsequent development of the QED-CASSCF methodology.

We use the following QED-CAS ansatz for the coupled electronic-photonic states:

$$\left|\Psi \right\rangle =\underset{M}{\sum }\;\underset{I}{\sum }C_{I,M}\left|\Phi_{I}^{e}\right\rangle ⊗\left|M^{p}\right\rangle$$ 2

where *C*_*I*,*M*_ is the expansion coefficient for a given product state composed of the *I*th electronic Slater determinant in the CAS expansion and a photonic Fock state with occupation number *M*. As in our prior work on QED-CASCI,^76^ we consider both the Pauli–Fierz Hamiltonian in eq 1 and its coherent state transformed counterpart, *Ĥ*_CS_ = *Û*_CS_ *Ĥ*_PF_ *Û*_CS_^†^, where *Û*_CS_ = exp­(*z*(*b̂*^†^ – *b̂*)) is the coherent state transformation defined by a parameter *z* = $-\frac{\left\langle \mathrm{d̂}\right\rangle }{\sqrt{2\omega }}$ that is determined at the mean-field (QED-RHF) level for all calculations.^29,95^ Because we may regard the photonic states in the latter case as |*M* ^CS^⟩ = *Û*_CS_^†^|*M* ^P^⟩, we will use the terminology of “photon number basis” when we use eq 2 as our ansatz for *Ĥ*_PF_, and “coherent state basis” when we use this ansatz for *Ĥ*_CS_. We will denote the approaches as CS-QED-CASSCF and PN-QED-CASSCF for the coherent-state and photon number bases, respectively.

In this work, we follow the “Uncoupled (CI)” algorithm described in ref 83. This algorithm is a variant of the Werner–Mayer–Knowles (WMK) method implemented in MOLPRO.^96^ The readers are referred to refs 81–84 for more details on the implementation for canonical electronic structure calculations. We use the letters *t*, *u*, *v*, and *w* to denote active orbitals, *i* and *j* to denote inactive orbitals, and *a*, *b* to represent virtual orbitals. Occupied orbitals are represented by *k*, *l*, *m*, *n*, and general orbitals are denoted with *p*, *q*, *r*, and *s*. We use the uppercase letter *M* to denote the occupation number for the Fock states of the cavity mode. The CAS Hamiltonian can be expressed on the photon-number basis as

$${Ĥ}_{\mathrm{PN}}^{\mathrm{CAS}}=\underset{tu}{\sum }F_{tu}^{c}{Ê}_{tu}+\frac{1}{2}\underset{tuvw}{\sum }{\left(tu|vw\right)}^{\prime }{Ê}_{tu,vw}-\sqrt{\frac{\omega }{2}}\left(\underset{tu}{\sum }d_{tu}{Ê}_{tu}+d_{n}+2\underset{i}{\sum }d_{ii}\right)\left({\mathrm{b̂}}^{†}+\mathrm{b̂}\right)+\omega {\mathrm{b̂}}^{†}\mathrm{b̂}+E_{c}^{\mathrm{PN}}$$ 3

and on the coherent-state transformed basis as

$${Ĥ}_{\mathrm{CS}}^{\mathrm{CAS}}=\underset{tu}{\sum }F_{tu}^{c}{Ê}_{tu}+\frac{1}{2}\underset{tuvw}{\sum }{\left(tu|vw\right)}^{\prime }{Ê}_{tu,vw}-\sqrt{\frac{\omega }{2}}\left(\underset{tu}{\sum }d_{tu}{Ê}_{tu}-\left\langle d_{e}\right\rangle +2\underset{i}{\sum }d_{ii}\right)\left({\mathrm{b̂}}^{†}+\mathrm{b̂}\right)+\omega {\mathrm{b̂}}^{†}\mathrm{b̂}+E_{c}^{\mathrm{CS}}$$ 4

Here, the operators *Ê*_*pq*_ and *Ê*_*pq*,*rs*_ are usual spin-free single and double excitation operators, respectively. The scalar quantities ⟨*d*_e_⟩ = **λ** · ⟨**μ**_*e*_⟩ and *d*_*n*_ = **λ** · **μ**_*n*_ are the field-scaled electronic dipole moment and nuclear dipole moment, and *d*_*pq*_ are field-scaled dipole integrals. Here we also assume that the term The modified-core Fock matrix and modified core electronic energy are given by

$$F_{rs}^{c}=h_{rs}^{\prime }+\underset{i}{\sum }\left[2{\left(rs|ii\right)}^{\prime }-{\left(ri|si\right)}^{\prime }\right]$$ 5

$$E_{c}^{\mathrm{PN}}=\underset{i}{\sum }\left[h_{ii}^{\prime }+{\left(F^{\prime }\right)}_{ii}\right]+\frac{1}{2}d_{n}^{2}$$ 6

$$E_{c}^{\mathrm{CS}}=\underset{i}{\sum }\left[h_{ii}^{\prime }+{\left(F^{\prime }\right)}_{ii}\right]+\frac{1}{2}{\left\langle d_{e}\right\rangle }^{2}$$ 7

The prime superscript indicates quantities that have been modified by contributions of the dipole self-energy, where we have slightly different modifications for the photon-number and coherent-state bases:

$$h_{pq}^{\prime }=h_{pq}-\frac{1}{2}q_{pq}+d_{n}d_{pq},(\mathrm{photon number basis})$$ 8

$$h_{pq}^{\prime }=h_{pq}-\frac{1}{2}q_{pq}-\langle \mathrm{d̂}\rangle_{e}d_{pq},\left(\mathrm{coherent state basis}\right)$$ 9

$${\left(pq|rs\right)}^{\prime }=\left(pq|rs\right)+d_{pq}d_{rs}$$ 10

Here, *q*_*pq*_ denotes the field-scaled quadrupole integrals.^24^

In the following, we present the adaptation of CASSCF theory to the coherent-state Pauli–Fierz Hamiltonian; that is, CS-QED-CASSCF. The analogous PN-QED-CASSCF follows a similar procedure. The CAS energy for the CS Hamiltonian is expressed in terms of the active reduced density matrices (RDMs) and wave function coefficients as

$$E_{\mathrm{CS}}^{\mathrm{CAS}}=E_{c}^{\mathrm{CS}}+\underset{IM}{\sum }M\omega {\left(C_{I,M}\right)}^{2}+\underset{tu}{\sum }F_{tu}^{c}D_{tu}+\underset{tuvw}{\sum }{\frac{1}{2}\left(tu|vw\right)}^{\prime }D_{tu,vw}-\sqrt{\frac{\omega }{2}}\underset{tu}{\sum }d_{tu}{\left(D_{pe}\right)}_{tu}+\sqrt{\frac{\omega }{2}}\left(\left\langle d_{e}\right\rangle -2\underset{i}{\sum }d_{ii}\right)\times \underset{IM}{\sum }\left(\sqrt{M}C_{I,M}C_{I,M-1}+\sqrt{M+1}C_{I,M}C_{I,M+1}\right)$$ 11

The 1- and 2- and photon–electron (pe)-RDMs are defined as

$$D_{pq}=\underset{IJM}{\sum }C_{I,M}C_{J,M}\left\langle I\right|{Ê}_{pq}\left|J\right\rangle$$ 12

$$D_{pq,rs}=\frac{1}{2}\underset{IJM}{\sum }C_{I,M}C_{J,M}\left\langle I\right|{Ê}_{pq,rs}+{Ê}_{qp,rs}\left|J\right\rangle$$ 13

$${\left(D_{\mathrm{pe}}\right)}_{pq}=\underset{IJM}{\sum }\left\langle I\right|{Ê}_{pq}\left|J\right\rangle \left(C_{I,M}C_{J,M-1}\sqrt{M}+C_{I,M}C_{J,M+1}\sqrt{M+1}\right)$$ 14

For convenience, we define these state-dependent scalar quantities:

$$X=\underset{IM}{\sum }M\omega {\left(C_{I,M}\right)}^{2}$$ 15

$$Y=\underset{IM}{\sum }\left(\sqrt{M}C_{I,M}C_{I,M-1}+\sqrt{M+1}C_{I,M}C_{I,M+1}\right)$$ 16

Here, the 2-RDM is symmetrized. In state-averaged CASSCF, the state-averaged CAS energy is expressed in terms of state-averaged RDMs. The active blocks of state-averaged RDMs are given by

$${\mathrm{D̅}}_{tu}=\underset{N}{\sum }W_{N}D_{tu}^{N}$$ 17

$${\mathrm{D̅}}_{tu,vw}=\underset{N}{\sum }W_{N}D_{tu,vw}^{N}$$ 18

$${\left({\mathrm{D̅}}_{\mathrm{pe}}\right)}_{tu}=\underset{N}{\sum }W_{N}{\left(D_{pe}^{N}\right)}_{tu}$$ 19

where *W*_*N*_ is the weight of the *N*th state (∑_*N*_ *W*_*N*_ = 1). Similarly, we can define these state-averaged quantities for eqs 15 and 16:

$$\mathrm{X̅}=\underset{N}{\sum }W_{N}\underset{IM}{\sum }M\omega {\left(C_{I,M}^{N}\right)}^{2}$$ 20

$$\mathrm{Y̅}=\underset{N}{\sum }W_{N}\underset{IM}{\sum }\left(\sqrt{M}C_{I,M}^{N}C_{I,M-1}^{N}+\sqrt{M+1}C_{I,M}^{N}C_{I,M+1}^{N}\right)$$ 21

The state-averaged CS-QED-CASSCF energy is then given by

$${\mathrm{E̅}}_{\mathrm{CS‐PF}}^{\mathrm{CAS}}=E_{c}^{\mathrm{CS}}+\mathrm{X̅}+\underset{tu}{\sum }F_{tu}^{c}{\mathrm{D̅}}_{tu}+\underset{tuvw}{\sum }{\frac{1}{2}\left(tu|vw\right)}^{\prime }{\mathrm{D̅}}_{tu,vw}-\sqrt{\frac{\omega }{2}}\underset{tu}{\sum }d_{tu}{\left({\mathrm{D̅}}_{\mathrm{pe}}\right)}_{tu}+\sqrt{\frac{\omega }{2}}\left(\left\langle d_{e}\right\rangle -2\underset{i}{\sum }d_{ii}\right)\mathrm{Y̅}$$ 22

In the Supporting Information, we describe our generalized second-order orbital optimization scheme that enables state-averaged QED-CASSCF calculations.

## Results

We perform illustrative calculations of the SA-QED-CASSCF approach on three model cavity-coupled systems: lithium hydride (LiH), hydroxide anion (OH^–^), and magnesium hydride cation (MgH^+^). To test these approaches against strong per-molecule coupling, we consider simulations where the fundamental coupling strength is in the range ∥**λ**∥∈ [0.01,0.05] atomic units, corresponding to cavity volumes between roughly 17 and 0.75 nm^3^, which is a domain that is feasible using plasmonic picocavities^26,27^ and scanning tunneling microscope break-junctions.^28^ We benchmark the QED-CASSCF results against QED-FCI for lithium hydride and hydroxide anion and against QED-DMRG for magnesium hydride cation. We see substantial improvements in the quality of the ground and polariton potential energy surfaces from QED-CASSCF over QED-CASCI in both cases, and we confirm the origin invariance of the total energy of the CS implementation of QED-CASSCF with both charged species. The canonical orbitals obtained from both PN- and CS-QED-CASSCF optimizations show origin dependence, as expected.^43,44^ Raw data in support of the results has been uploaded to the Zenodo open research repository, and can be found in ref 97.

### Lithium Hydride

First, we consider the LiH molecule coupled to a cavity mode polarized along the internuclear axis λ_*z*_ = 0.05 au to couple to the *S*_0_ → *S*_1_ transition at a bond length of *r* ≈ 1.7 Å with *ℏ*ω = 3.29 eV. We represent the LiH molecule in a 6–311G basis set^98^ for the QED-CASSCF, QED-CASCI, and QED-FCI calculations, and represent the photonic Fock space in a basis of coherent states, *Û*_CS_|0⟩, *Û*_CS_|1⟩, ..., *Û*_CS_|*N*_ph_⟩ (where *N*_ph_ denotes the maximum occupation state). We use the nomenclature (*N*_e_,*N*_o_,*N*_ph_) to denote the number of active electrons, orbitals, and maximum occupation state, respectively, in a given calculation. For SA-QED-CASSCF calculations, we consider state averaging over the first three singlet states with equal weight, corresponding to the ground, lower-polariton, and upper-polariton states. Equal-weight state-averaged calculations provide a balanced treatment of all states included within the SA-CASSCF ensemble. It has been noted in the literature that, in cases of conical intersections, discontinuities in the potential energy surface can occur if degenerate states are not treated on equal footing.^99^ Given that the polariton states result from degeneracies between uncoupled states (e.g., cases where the energy of *S*_0_ + *ℏ*ω = *S*_1_) and often leads to near-degeneracies of the resulting coupled states, we find it prudent to consider equal weighting as a first step. The use of unequal-weight SA-CASSCF methods can lead to numerical instabilities in the SCF optimization process, as contributions from low-weight states become negligible in determining the orbitals while their wave functions still require optimization. Although dynamically weighted SA-CASSCF approaches are possible and may offer improvements,^99^ they introduce additional parameters that require optimization, which is beyond the scope of this work.

We first take a broad scan of the performance of SA-QED-CASSCF compared to QED-CASCI for the coupled ground state by systematically increasing the active space size and computing the nonparallelity error (NPE) across the LiH ground-state potential energy surface (between 1.0 and 3.0 Å). We correlate all 4 electrons and consider a minimal (4_e_,4_o_,1_ph_) active space up to a (4_e_,15_o_,1_ph_) active space by incrementally adding additional virtual orbitals (see Figure 1). We compute the QED-CASCI­(4_e_,*N*_o_,1_ph_) and QED-CASSCF­(4_e_,*N*_o_,1_ph_) NPEs relative to QED-FCI, which is equivalent to a (4_e_,16_o_,1_ph_) active space. We see that the QED-CASSCF ground-state surfaces show consistently smaller NPEs, typically an order of magnitude lower than that of QED-CASCI for the same active space. In particular, the SA-QED-CASSCF NPE falls below 1 kcal/mol (“chemical accuracy”) by the (4e,8o), whereas a (4e,15o) active space is required for QED-CASCI to fall under this threshold. We note that this systematic increase in virtual orbital size does not necessarily improve the quality of the active space at each increment, since there will be, for example, pairs of degenerate orbitals that will not be properly included at certain increments. This can be seen in the nonmonotonic improvement in the QED-CASCI NPEs with increasing active orbital sizes. We see that QED-CASSCF optimization at least partially alleviates this issue, with the QED-CASSCF showing closer to monotonically decreasing NPE with increasing active space size.

**Figure 1:**
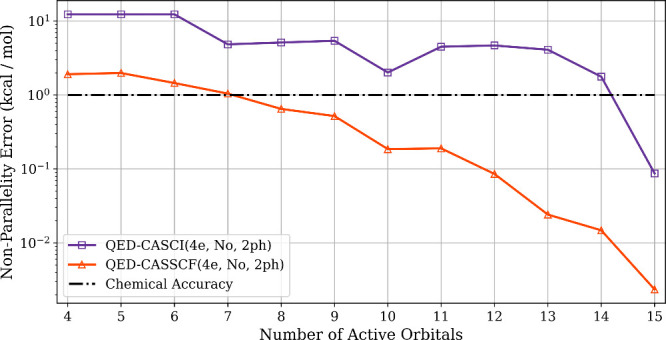
Nonparallelity errors (NPE) in kcal/mol of the ground-state potential-energy curves of LiH coupled to a cavity as a function of active space size for both QED-CASCI and QED-CASSCF.

Next, we turn our attention to the performance of SA-QED-CASSCF on the ground and polariton states of LiH under the same coupling conditions. Here we focus on an active space of (4_e_,9_o_,10_ph_) for all CAS calculations, and for the SA-QED-CASSCF, we consider equal weighting of the ground, lower-polariton, and upper-polariton states.

In Figure 2, we show the ground, lower polariton, and upper polariton potential energy surfaces with fixed active space sizes of (4_e_,9_o_,10_ph_) at the QED-CASCI and SA-QED-CASSCF levels compared to the QED-FCI surfaces. We see that the QED-CASCI ground state surface qualitatively matches the QED-FCI curve for all states, but we also observe it is substantially higher in energy, suggesting that it is missing a large amount of the total correlation energy (see Figure 2). The SA-QED-CASSCF ground-state curve matches the shape of the QED-FCI curve as well, and comes much closer to matching the curve in absolute energy, suggesting that the QED-CASSCF procedure can help to recover a substantial amount of correlation. We see that the SA-QED-CASSCF curves continue to provide a close qualitative and quantitative match to the lower- and upper-polariton curves but that the QED-CASCI suffers both qualitatively and quantitatively for this active space (see Figure 2). For the lower-polariton surface, we see that the QED-CASCI predicts a minimum at a shorter bond length: QED-CASCI predicts a minimum at *r* ≈ 1.6 Å vs the SA-QED-CASSCF and QED-FCI curves that predict a minimum at *r* ≈ 1.8 Å (see Figure 2). The upper-polariton surface, as computed by all levels, shows a change in behavior resulting from an interaction (an apparent avoided crossing) with a higher-lying state, which is also coupled to the upper polariton state through the bilinear coupling. However, the location of this feature in the PES occurs at *r* ≈ 2.25 Å in the QED-FCI and SA-QED-CASSCF curves, but closer to *r* ≈ 2.4 Å at the QED-CASCI level (see Figure 2). Thus, we see that the SA-QED-CASSCF procedure provides the potential to add both qualitative and quantitative accuracy to the potential energy surfaces of states under strong coupling. We specifically focus on the (4_e_,9_o_) active space because we find it capable of capturing this interaction feature in the upper-polariton surface. In Figure S1, we show an illustrative calculation where QED-CASCI­(4_e_,8_o_,10_ph_) does not capture this feature of the upper-polariton surface. We note that these features can be quite difficult to resolve numerically, and other numerical parameters like the size of the subspace used in the iterative eigensolver for the CASCI step can impact the convergence. We plan to explore these numerical features in more detail in future studies.

**Figure 2:**
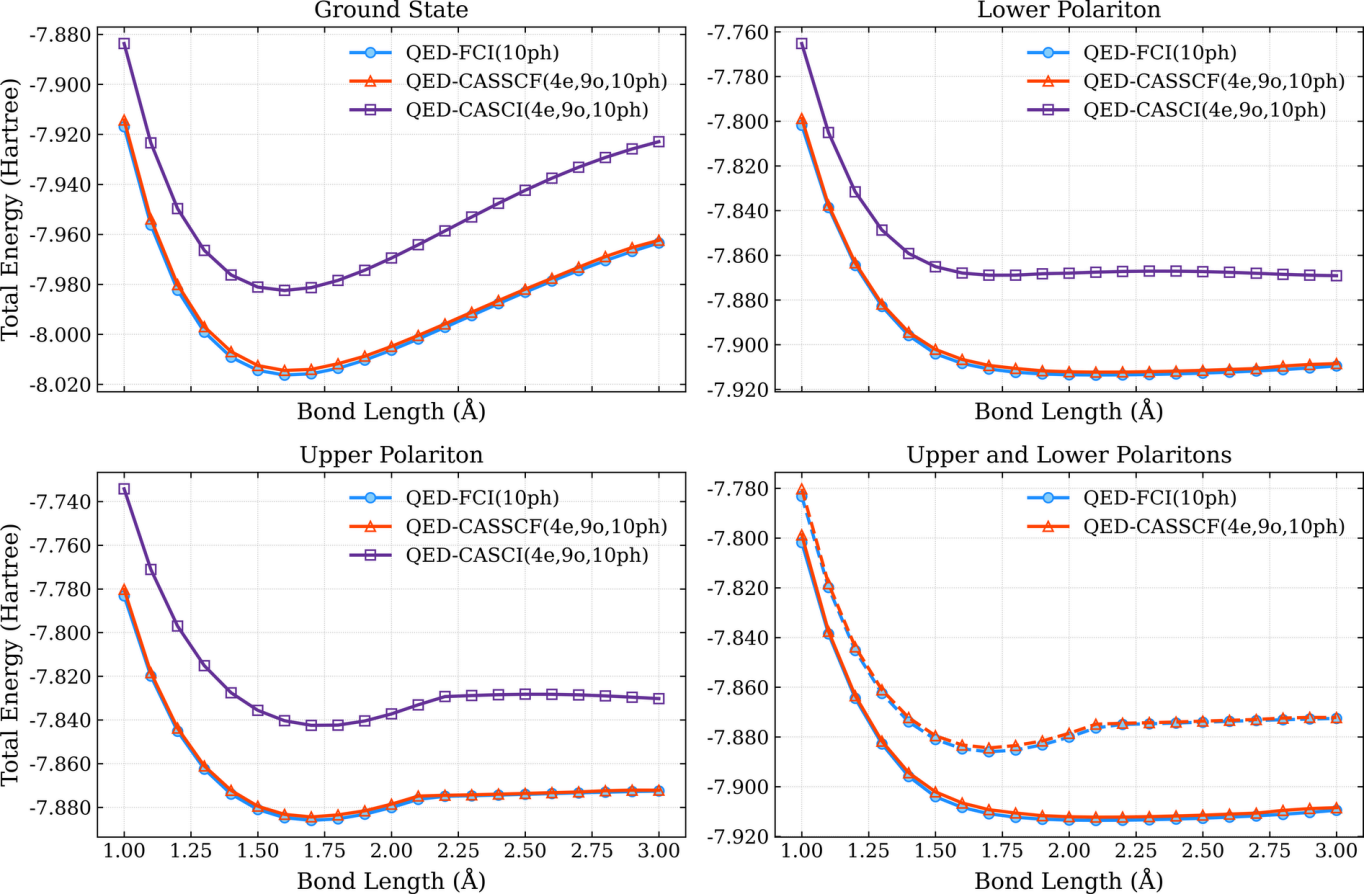
Ground state (top left), lower polariton (top right), and upper polariton (lower left) potential energy surfaces for the LiH molecule computed using QED-CASCI­(4_e_,9_o_,10_ph_) SA-QED-CASSCF­(4_e_,9_o_,10_ph_), and QED-FCI­(10_ph_) all within a 6–311G basis set. The upper and lower polariton surfaces are shown together at the SA-QED-CASSCF­(4_e_,9_o_,10_ph_), and QED-FCI­(10_ph_) levels in the bottom right panel.

As shown in Table 1, the mean absolute errors (MAEs) for all QED-CASSCF surfaces are at or below ∼1 kcal/mol, whereas the MAES of the QED-CASCI surfaces all exceed 20 kcal/mol. It is interesting to note that the MAE for the QED-CASSCF ground state is slightly larger than that for the polariton states. We did not make any effort to optimize the state weighting, and it is likely that greater weighting to the ground-state in state averaging could improve this MAE further.

**Table 1:** Mean Absolute Errors (MAEs) between QED-CASCI­(4_e_,9_o_,10_ph_)/QED-CASSCF­(4_e_,9_o_,10_ph_) and QED-FCI­(10_ph_) Reference Energies for Three Polaritonic States, All in a 6-311G Basis Set

	Mean Absolute Error, MAE(kcal/mol)	
state	QED-CASCI(4_e_,9_o_,10_ph_)	SA-QED-CASSCF(4_e_,9_o_,10_ph_)
ground	23.181	1.055
lower polariton	26.059	0.905
upper polariton	28.326	0.723

### Magnesium Hydride Cation

Next, we consider the (MgH^+^) cation coupled to a cavity mode polarized along the internuclear axis with two coupling strengths, λ_*z*_ = 0.01 and λ_*z*_ = 0.05 au, to couple to the *S*_0_ → *S*_1_ transition at a bond length of *r* ≈ 2.2 Å with *ℏ*ω = 3.70 eV. For all calculations on this system, we perform state averaging with equal weighting of the first three singlet states. These correspond to the ground state, lower polariton, and upper polariton states for the cavity-coupled system and the *S*_0_, *S*_1_, and *S*_2_ states for the cavity-free system; note only the *S*_0_ and *S*_1_ states are reported on in the cavity-free case in Figure 3 and Table 2. For all CAS calculations, we use an active space of (8e,12o), we represent the magnesium hydride cation in the cc-pVDZ basis set,^100^ and represent the photonic Fock space using the coherent states with *N*_p_ = 10 for all cavity-coupled potential energy surface calculations. We leverage our recent QED-DMRG implementation to approximate the QED-FCI solutions by correlating all electrons into all orbitals (12e,23o), which serves as our ground truth for this study. The approximation inherent in the QED-DMRG calculations arises from the truncation of the bond dimension of the matrix product state.^77^ For all calculations, we truncate the bond dimension at 1000, which is sufficient to yield truncation errors in the average energy of *S*_0_, LP, and UP at the micro-Hartree level (see Figure S2 for a plot of the maximum truncation error as a function of bond dimension).

**Figure 3:**
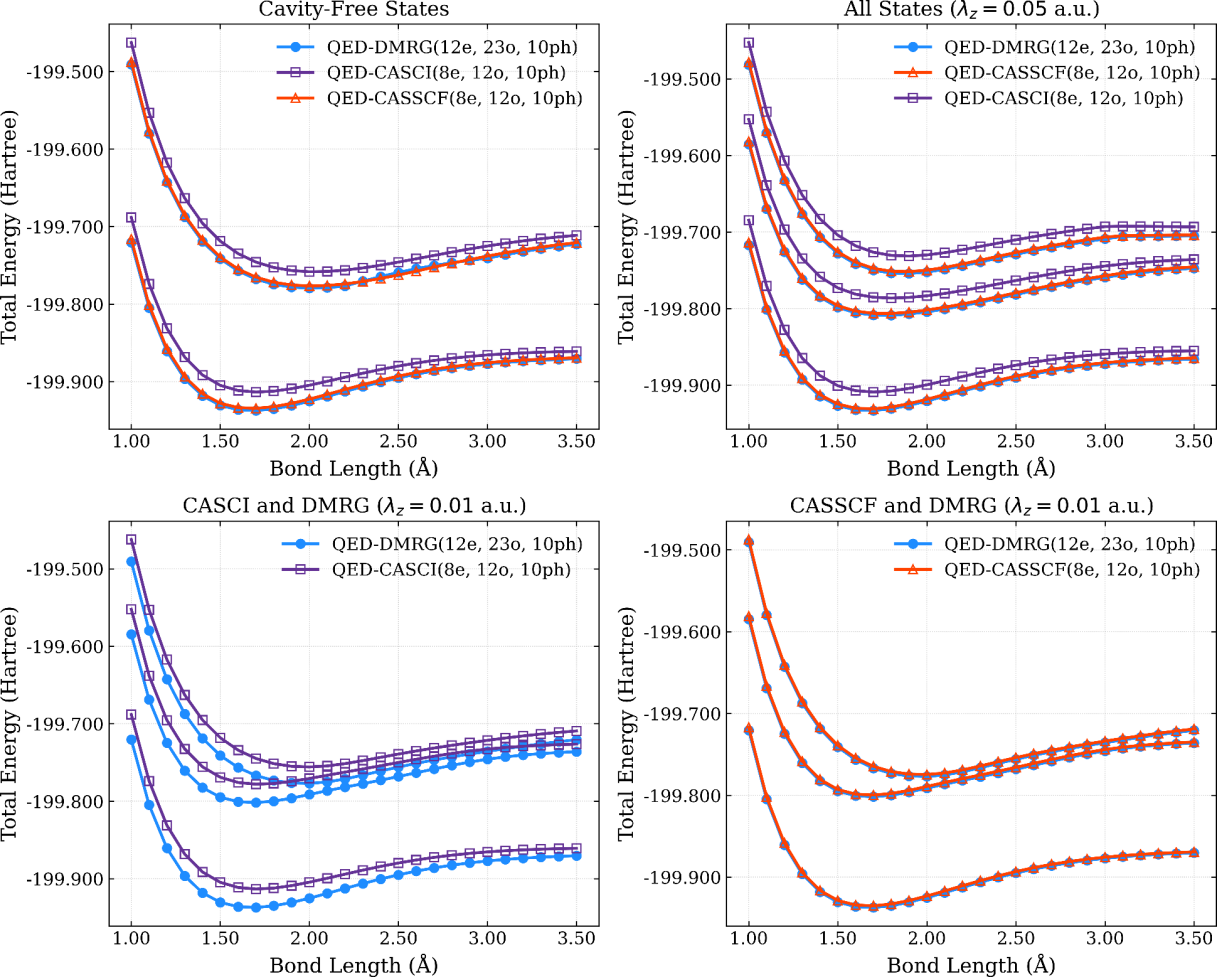
Ground and excited-state surfaces from QED-DMRG, QED-CASSCF, and QED-CASCI under different coupling conditions, including no coupling (top-left), resonant coupling with λ_*z*_ = 0.05 au (top-right), and resonant coupling with λ_*z*_ = 0.01 au (bottom panels).

**Table 2:** Mean Absolute Errors (MAEs) between QED-CASCI/QED-CASSCF and QED-DMRG Reference Energies under Different Cavity Coupling Conditions

	Mean Absolute Error, MAE (kcal/mol)	
state	QED-CASCI(8_e_,12_o_,10_ph_)	SA-QED-CASSCF(8_e_,12_o_,10_ph_)
**Cavity-free**		
S_0_	11.825	1.519
S_1_	11.620	1.485
**λ_*z*_ = 0.01 au**		
ground	11.840	1.193
lower polariton	12.376	1.309
upper polariton	11.883	1.356
**λ_*z*_ = 0.05 au**		
ground	12.316	1.305
lower polariton	12.692	1.447
upper polariton	12.617	1.385

In Figures 3, we show the potential energy surfaces from all methods under the different coupling conditions, as well as the cavity-free surfaces. In all cases, we see excellent agreement between the QED-CASSCF surfaces and the QED-DMRG surfaces, with these surfaces nearly overlapping, despite the exclusion of 4 core electrons and 11 virtual orbitals from the (8e,12o) active space used in the CASSCF and QED-CASSCF calculations. We again see that there are visible quantitative differences between QED-CASCI and QED-DMRG surfaces in all cases. Interestingly, these quantitative errors in QED-CASCI are significant enough in the λ_*z*_ = 0.01 case such that the QED-CASCI lower polariton surface is higher in energy than the QED-DMRG upper polariton surface for bond lengths between *r* ≈ 1.8 and 3.0 Å. We compute the mean absolute error for all QED-CASCI and SA-QED-CASSCF surfaces within this (8_e_,12_o_,10_ph_) active space relative to the QED-DMRG reference and find that the mean absolute errors are roughly ten times larger for all QED-CASCI surfaces (between 11 and 12 kcal/mol), whereas the mean absolute errors for all QED-CASSCF surfaces are between 1.1 and 1.5 kcal/mol (see Table 2).

### Origin Invariance

The QED formulation of ab initio methods employing the PF Hamiltonian exhibits origin dependence in charged molecular systems due to the interplay between the dipole operator inherent to the PF framework and the finite photonic Fock space.^95^ This limitation is mitigated by either exhausting the photonic Fock space or transforming the PF Hamiltonian, for example, with coherent state transformation, which restores numerical origin invariance in QED-HF, QED-CC, and QED-FCI energies. In this work, we demonstrate that the SA-CS-CASSCF approach similarly achieves origin invariance for both state-averaged and individual state energies.

As a first example, we examine the ground-state energy error of the hydroxide anion (OH^–^) computed with QED-CASSCF when the molecular center is displaced by 20 Å along the *z*-axis from the cavity origin, compared to the energy obtained with the molecule at the origin (Figure 4). Calculations were performed using two different active spaces, (2e,2o) and (6e,6o), and various photonic Fock space truncations (1, 5, 20, and 50 photons), employing both the standard Pauli–Fierz (PN) and the CS formulations of the Hamiltonian. All computations used the 6–31G basis set,^101^ with the O–H bond length fixed at 0.9 Å The cavity parameters were set to *ℏ*ω = 5.96 eV and λ_*z*_ = 0.05 au, with the coupling vector aligned along the molecular *z*-axis. For both active spaces considered, the PN-QED-CASSCF energies exhibit a marked origin dependence, with energy errors on the order of 1.0 *E*_h_ even when in a minimal photonic Fock space (*N*_ph_ = 1), and an error of roughly 0.35 *E*_h_ when *N*_ph_ = 5. In contrast, the CS-QED-CASSCF energies are numerically origin invariant, yielding errors as small as 1.0 × 10^–12^ *E*_h_ for both active spaces, even in a minimal photonic Fock space. These results corroborate prior results showing the a state-specific QED-CASSCF approach yields origin invariant energies under coherent state transformation.^80^

**Figure 4:**
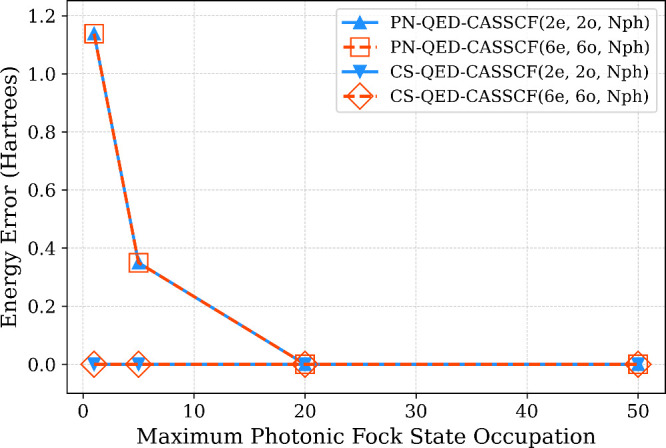
Origin-dependent error for the OH^–^ anion as a function of the number of photonic Fock states for the CS and PN basis.

As discussed in refs 43 and 44 , even though total energies resulting from SCF procedures subject to the coherent-state transformation (e.g., CS-QED-HF) are origin-invariant, the CS-transformed Fock matrix is not origin-invariant for charged species, and so the canonical orbitals for charged species are also not origin-invariant. The same is true for the CS-QED-CASSCF approach, as can be seen from the canonical CS-QED-CASSCF orbitals of OH^–^ spanning the (6e,6o) active space at the origin (0,0,0) and under displacement (0,0,20) (see the top panel of Figure 5, orbitals that display visible origin dependence are boxed in red). For comparison, we also show the PN-QED-CASSCF canonical orbitals for OH^–^ at the origin and under displacement in the bottom panel of Figure 5. We further present the CS-QED-CASSCF and PN-QED-CASSCF natural orbitals and occupation numbers spanning the (6e,6o) active space in Figure 6. We find that the PN-QED-CASSCF natural orbitals and their occupation numbers have origin dependence; we also box the natural orbitals that show a visible origin dependence in red to aid visualization. By contrast, the natural orbital occupation numbers from CS-QED-CASSCF are found to be origin invariant, and we do not discern a visible origin dependence in these natural orbitals.

**Figure 5:**
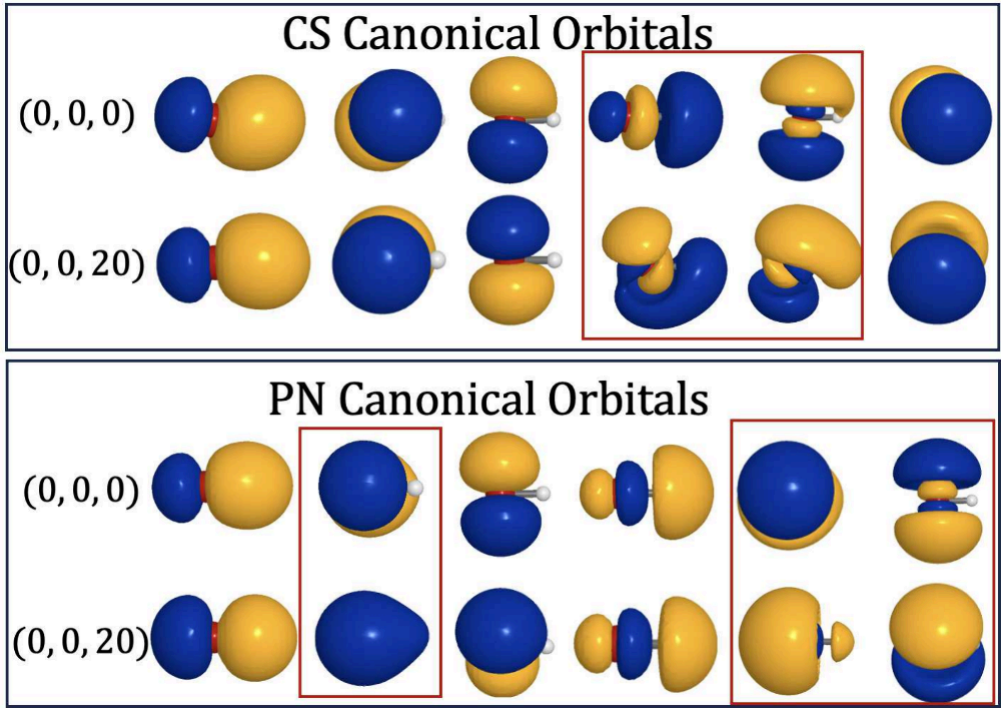
Canonical orbitals from the active space of the CS-QED-CASSCF­(6e,6e,1ph) (top) and PN-QED-CASSCF­(6e,6o,1ph) (bottom) calculations of OH^–^ at the origin (0,0,0) and under displacement by 20 Å­(0,0,20). Orbitals which display visible origin dependence are boxed in red.

**Figure 6:**
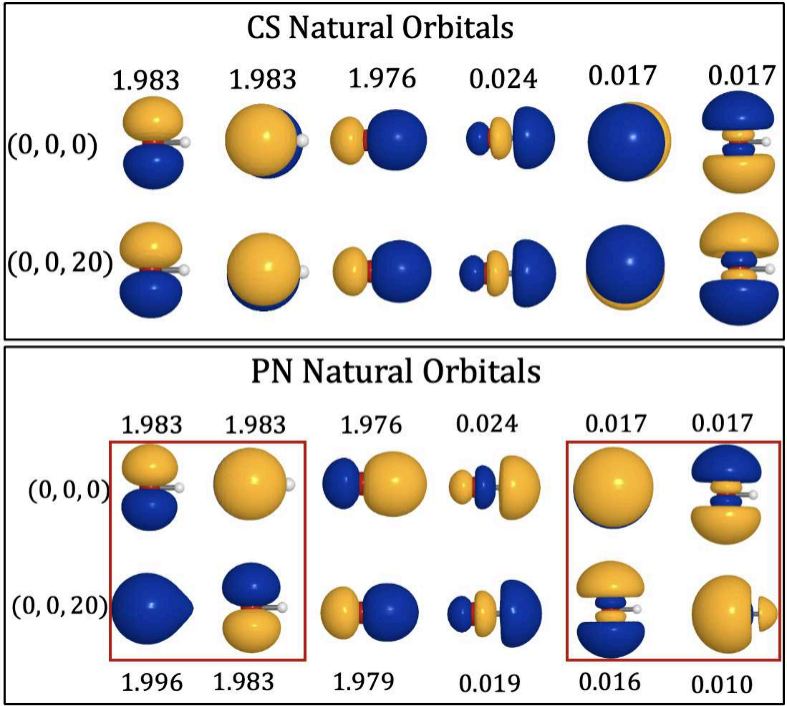
Natural orbitals and occupation numbers from the active space of the CS-QED-CASSCF­(6e,6e,1ph) (top) and PN-QED-CASSCF­(6e,6o,1ph) (bottom) calculations of OH^–^ at the origin (0,0,0) and under displacement by 20 Å­(0,0,20). Orbitals displaying visible origin dependence are boxed in red.

As a final case study, we analyze the origin invariance of the first three singlet states of MgH^+^ computed via state-averaged QED-CASSCF (SA-QED-CASSCF), comparing energies for the molecule centered at the origin versus displaced by 20 Å along the *z*-axis (Figure 7). Here, we used the same basis set, active space, and cavity frequency as that which was employed using our previous PES scan for MgH^+^, and we fixed the bond length at 2.0 Å and λ_*z*_ = 0.05 au, with the coupling vector aligned along the molecular *z*-axis. Photonic Fock space truncations of *N*_ph_ = 1, 5, and 10 were investigated. The results demonstrate numerical origin invariance for the state-averaged energy (Δ*E*_avg_ ≈ 1.0 × 10^–11^ *E*_h_) across all *N*_ph_ values. However, individual state energies exhibit larger deviations (Δ*E*_indiv_ ≈ 1.0 × 10^–8^ *E*_h_). This discrepancy arises because the SA-QED-CASSCF optimization prioritizes minimization of the state-averaged energy rather than individual state energies. To confirm this interpretation, we repeated calculations exclusively at the origin geometry, observing consistent Δ*E*_avg_ < 1.0 × 10^–11^ *E*_h_, while Δ*E*_indiv_ remained ∼1.0 × 10^–8^ *E*_h_. These findings underscore that origin invariance in SA-QED-CASSCF is rigorously maintained for the state-averaged observable but manifests larger numerical fluctuations in individual states due to the optimization constraints.

**Figure 7:**
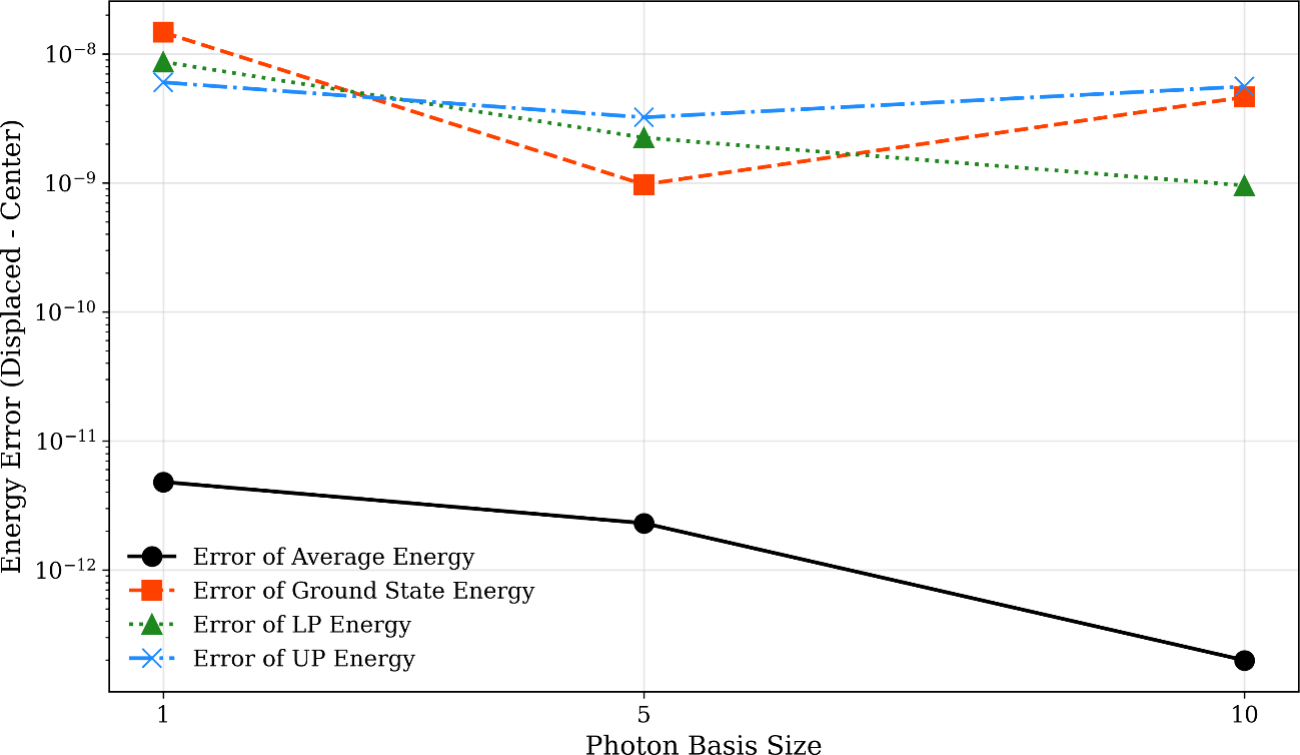
Origin-dependent error for the MgH^+^ cation as a function of the number of photonic Fock states.

## Conclusion

In this work, we have presented a generalization of the state-averaged complete active space self-consistent field (CASSCF) theory for ground and excited states of strongly coupled light–matter systems. We specifically adapted the WMK second-order optimization scheme with a model function that remains infinite-order in orbital rotation parameters, facilitating more robust convergence. In addition to detailing the theory, implementation, and numerical challenges, we demonstrated this approach using simple yet illustrative systems exhibiting strong light-matter coupling. These demonstrations revealed significant advantages of the SA-QED-CASSCF method over the existing QED-CASCI methodology in predicting polaritonic potential energy surfaces, achieving strong qualitative and quantitative agreement with QED-FCI results. We anticipate this methodology will facilitate detailed studies QED Chemistry where strong multireference correlation that arises from strained geometries, long-range cavity-induced correlations, and/or from the mixing of many electronic excited-states may be relevant. As one concrete example, we believe this can be part of a valuable toolkit for corroborating the potential for ground-state modification of nitrobenzene,^8^ where LR-TDDFT was used to parametrize a projected Pauli–Fierz model with roughly 50 electronic states. The extent to which multireference correlation effects are a factor in this enticing cavity-modified chemistry remains to be seen.

## Supplementary Material



## Data Availability

The implementation of the QED-CASSCF method used for the results presented within can be accessed in the following GitHub repository:https://github.com/mapol-chem/qed-ci/tree/casscf. Raw data in support of the results has been uploaded to the Zenodo open research repository, and can be found here: 10.5281/zenodo.15595754.

## Notes

Representative polaritonic potential energy surface with a suitable and smaller-than-suitable active space (Figure S1); maximum error vs bond dimension for QED-DMRG (Figure S2); additional details on the orbital optimization scheme for SA-QED-CASSCF (PDF)
