# Supplementary material for: Modeling Strong Light-Matter Coupling in Correlated Systems: State-Averaged Cavity Quantum Electrodynamics Complete Active Space Self-Consistent Field Theory
Source: J Chem Theory Comput. 2025 Aug 30;21(18):8812–22. doi: 10.1021/acs.jctc.5c00927 (PMC12461917; doi:10.1021/acs.jctc.5c00927)
Supplement: Supplementary file 1 [file ct5c00927_si_001.pdf]

Supporting Information for: Modeling Strong Light-Matter Coupling in Correlated Systems: State-Averaged Cavity Quantum Electrodynamics Complete Active Space Self-Consistent Field Theory

Nam Vu,[*,†] Kenny Ampoh,[†] Mikuláš Matoušek,[‡,¶] Libor Veis,[‡] Niranjan Govind,[§,‖] and Jonathan J. Foley IV[*,†]

†*Department of Chemistry, University of North Carolina Charlotte, Charlotte, North Carolina 28223, USA*

‡*J. Heyrovský Institute of Physical Chemistry, Academy of Sciences of the Czech Republic, v.v.i., Dolejškova 3, 18223 Prague 8, Czech Republic*

¶*Faculty of Mathematics and Physics, Charles University, 12116 Prague 2, Czech Republic*

§*Physical and Computational Sciences Directorate, Pacific Northwest National Laboratory, Richland, Washington 99352, United States*

‖*Department of Chemistry, University of Washington, Seattle, Washington 98195, United States*

E-mail: nvu12@charlotte.edu; jfoley19@charlotte.edu

Supporting Figures

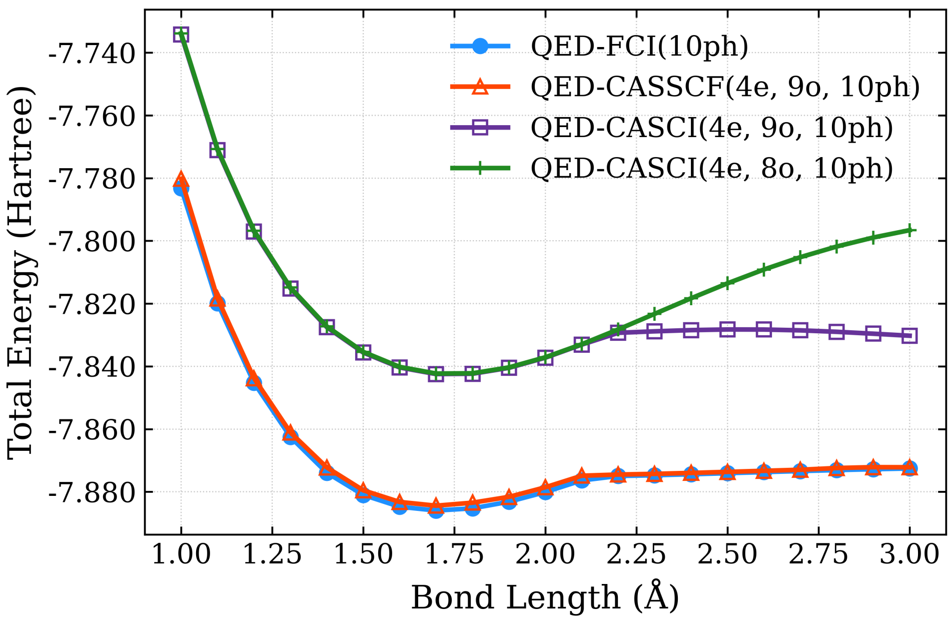

Figure S1: The upper polariton potential energy surfaces for the LiH molecule computed using QED-CASCI$(4_e, 8_o, 10_{ph})$, QED-CASCI$(4_e, 9_o, 10_{ph})$ SA-QED-CASSCF$(4_e, 9_o, 10_{ph})$, and QED-FCI(10_{ph}) all within a 6-311G basis set. We see that the $(4_e, 8_o, 10_{ph})$ active space cannot qualitatively capture the features of the upper polariton surface at long bond lengths.

QED-CASSCF Procedure

In the following discussion, the labels for state-averaged quantities will be omitted because all the expressions for a single state are identical for the averaged state. Recalling from the main text (Eq.

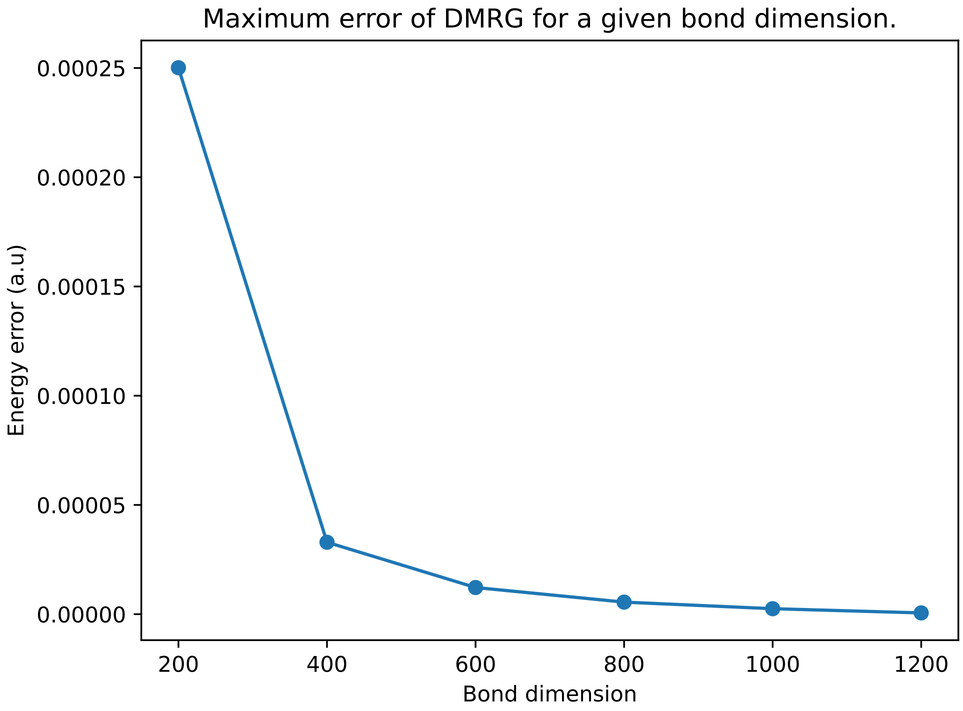

Figure S2: The maximum truncation error as a function of bond dimension of the QED-DMRG method. This is defined as the largest truncation error from calculation of the ground-state, lower-, and upper-polariton energies for the MgH^+ system at $r = 1.7$ Angstroms with $\lambda_z = 0.05$ a.u. and $\hbar\omega = 3.70$ eV.

22) that the CS-QED-CASSCF energy can be written as:

$$\begin{aligned}\bar{E}^{CAS}_{CS-PF} = {} & E^{CS}_{c} + \bar{X} \\ & + \sum_{tu} F^{c}_{tu}\bar{D}_{tu} + \sum_{tuvw} \frac{1}{2}(tu|vw)'\bar{D}_{tu,vw} \\ & - \sqrt{\frac{\omega}{2}} \sum_{tu} d_{tu}(\bar{D}_{\text{pe}})_{tu} \\ & + \sqrt{\frac{\omega}{2}} (\langle d_e \rangle - 2\sum_{i} d_{ii})\bar{Y}. \end{aligned} \tag{S1}$$

We seek to find an optimal orbital basis to optimize the state averaged energy.

In a rotated orbital basis, the one- ($\mathbf{h}', \mathbf{d}$) and two-electron integrals $\mathbf{g}'$ are transformed as:

$$\mathbf{h}' \rightarrow \mathbf{U}^T \mathbf{h}' \mathbf{U} \tag{S2}$$

$$\mathbf{d} \rightarrow \mathbf{U}^T \mathbf{d} \mathbf{U} \tag{S3}$$

$$\mathbf{g}' \rightarrow \mathbf{U}^T \mathbf{U}^T \mathbf{g}' \mathbf{U} \mathbf{U} \tag{S4}$$

Therefore, the total energy is a fourth power function of the orbital rotation matrix $\mathbf{U}$, which we denote $E(\mathbf{U})$. By writing $\mathbf{U} = \mathbf{1} + \mathbf{T}$ and truncating $E(\mathbf{T})$ at second order in $\mathbf{T}$ we get:

$$E^{(2)}(\mathbf{T}) = E_0 + 2\sum_{rk} T_{rk}A_{rk} + \sum_{klrs} T_{rk}G^{kl}_{rs}T_{sl} \tag{S5}$$

where $E_0 = E(\mathbf{U} = \mathbf{1})$. In the WMK method, we optimize $E^{(2)}(\mathbf{T})$ until $E(\mathbf{U})$ is self-consistent. The $E^{(2)}(\mathbf{T})$ model is a good approximation for the exact energy since $\mathbf{T}$, similar to $\mathbf{U}$, is periodic. The evaluation of energy in this model only requires a two-index integral transformation, therefore, the number of four-index transformations can be greatly reduced.

The matrix **A** is given by:

$$A_{ri} = 2F_{ri} - 2\sqrt{\frac{\omega}{2}}d_{ri}Y \tag{S6}$$

$$A_{ru} = \sum_t F^{\mathrm{c}}_{rt}D_{tu} + \sum_{tvw}(rt|vw)'D_{tu,vw} - \sum_t \sqrt{\frac{\omega}{2}}d_{rt}(D_{\mathrm{pe}})_{ut} \tag{S7}$$

where we define the intermediates:

$$J^{kl}_{rs} = (rs|kl)' \tag{S8}$$

$$K^{kl}_{rs} = (rk|sl)' \tag{S9}$$

$$F^{c}_{rs} = h'_{rs} + \sum_j \left[2(rs|jj)' - (rj|js)'\right] \tag{S10}$$

$$F_{rs} = F^{c}_{rs} + \sum_{tu} D_{tu}\left[J^{tu}_{rs} - \frac{1}{2}K^{tu}_{rs}\right] \tag{S11}$$

$$L^{kj}_{rs} = 4K^{kj}_{rs} - K^{kj}_{sr} - J^{kj}_{rs}. \tag{S12}$$

The matrix **G** is given by:

$$G^{ij}_{rs} = 2(F_{rs}\delta_{ij} + L^{ij}_{rs}) - 2\sqrt{\frac{\omega}{2}}d_{rs}\delta_{ij}Y \tag{S13}$$

$$G^{tj}_{rs} = \sum_v D_{tv}L^{vj}_{rs} = G^{jt}_{sr} \tag{S14}$$

$$G^{tu}_{rs} = F^{c}_{rs}D_{tu} + \sum_{vw}[J^{vw}_{rs}D_{tu,vw} + 2K^{vw}_{rs}D_{tv,uw}] - \sqrt{\frac{\omega}{2}}d_{rs}(D_{\mathrm{pe}})_{tu} \tag{S15}$$

To apply a second-order optimization for $E^{(2)}(\mathbf{T})$, we define an update of $\mathbf{T}$ at a point $\mathbf{T}_0 = \mathbf{T}(\mathbf{R}_0)$ as $\mathbf{T}(\mathbf{R}_0, \mathbf{R})$ and:

$$\mathbf{T}(\mathbf{R}_0, \mathbf{R}) = \mathbf{T}(\mathbf{R}_0) + \Delta\mathbf{T} \tag{S16}$$

$$\mathbf{T}(\mathbf{R}_0) = \mathbf{U}(\mathbf{R}_0) - \mathbf{1} \tag{S17}$$

$$\Delta\mathbf{T} = \mathbf{U}(\mathbf{R}_0)(\mathbf{R} + \frac{1}{2}\mathbf{R}^2 + ...) \tag{S18}$$

Inserting Eq. S18 into Eq. S5, truncating the energy expression at the second-order in R gives:

$$E^{(2)}(\mathbf{T}_0,\mathbf{R}) = E^{(2)}(\mathbf{T}_0) + 2\sum_{rk}\tilde{A}_{rk}\left[R_{rk} + \frac{1}{2}(\boldsymbol{R}^2)_{rk}\right] + \sum_{klrs} R_{rk}\tilde{G}^{kl}_{rs}R_{sl} \tag{S19}$$

where the matrix $\tilde{A}, \tilde{G}$ are given by:

$$\tilde{A}_{rk} = (\mathbf{U}(\mathbf{R}_0)^T\mathbf{B})_{rk}, \quad \tilde{A}_{ra} = 0 \tag{S20}$$

$$B_{rk} = A_{rk} + \sum_{sl} G^{kl}_{rs}T_{sl}, \quad B_{ra} = 0 \tag{S21}$$

$$\tilde{G}^{kl}_{rs} = (\mathbf{U}(\mathbf{R}_0)^T\mathbf{G}^{kl}\mathbf{U}(\mathbf{R}_0))_{rs} \tag{S22}$$

Taking the first and second partial derivative of $E^{(2)}(\mathbf{T}_0,\mathbf{R})$ with respect to $\mathbf{R}$ while taking into account that the derivatives must satisfy the same antisymmetry constraint of $\mathbf{R}$ and the second derivative is symmetric, i.e. $\frac{\partial^2 E}{\partial R_{rk}\partial R_{sl}}\big|_{\mathbf{R}=0} = \frac{\partial^2 E}{\partial R_{sl}\partial R_{rk}}\big|_{\mathbf{R}=0}$ provides us the gradient $\tilde{\mathbf{a}}$ and Hessian $\tilde{\mathbf{H}}$:

$$\tilde{a}_{rk} = 2(\tilde{A}_{rk} - \tilde{A}_{kr}) \tag{S23}$$

$$\tilde{H}_{rk,sl} = (1-\hat{P}_{rk})(1-\hat{P}_{sl})\left[2\tilde{G}^{kl}_{rs} - \delta_{kl}(\tilde{A}_{rs} + \tilde{A}_{sr})\right] \tag{S24}$$

Thus, the orbital optimization of the $E^{(2)}(\mathbf{T})$ model is equivalent to finding the unitary matrix $\mathbf{U}$ that satisfies the condition $\tilde{a}_{rk} = 0$. Following orbital optimization, a second-order orbital transformation is performed where the second-order updates of core energy and integrals are expressed

as:

$$E_c^{(2)} = E_c^{CS} + 4\sum_i (\mathbf{F}^c\mathbf{T})_{ii} + 2\sum_{ij}\left[\mathbf{T}^T(\mathbf{F^c}\delta_{ij} + \mathbf{L}^{ij})\mathbf{T}\right]_{ij} \tag{S25}$$

$$F_{tu}^{c(2)} = (\mathbf{U}^T\mathbf{F}^c\mathbf{U})_{tu} + \sum_i \left[2(\mathbf{U}^T\mathbf{J}^{tu}\mathbf{U} - \mathbf{J}^{tu})_{ii} - 2(\mathbf{U}^T\mathbf{K}^{tu}\mathbf{U} - \mathbf{K}^{tu})_{ii} + (\mathbf{T}^T\mathbf{L}^{ui}\mathbf{T})_{ti} + (\mathbf{T}^T\mathbf{L}^{ti}\mathbf{T})_{ui}\right] \tag{S26}$$

$$(tu|vw)'^{(2)} = -(tu|vw)' + (\mathbf{U}^T\mathbf{J}^{vw}\mathbf{U}_{tu} + (\mathbf{U}^T\mathbf{J}^{tu}\mathbf{U})_{vw} + (1+\hat{P}_{tu})(1+\hat{P}_{vw})(\mathbf{T}^T\mathbf{K}^{tv}\mathbf{T})_{uw} \tag{S27}$$

and subsequently a second-order CI calculation is performed. These steps constitute a microiteration in the WMK method. The second-order CAS CS-PF operator is given by:

$$\hat{H}_{CS-PF}^{CAS(2)} = E_c^{(2)} + \omega\hat{b}^\dagger\hat{b} + \sum_{tu} F_{tu}^{c(2)}\hat{E}_{tu} + \frac{1}{2}\sum_{tuvw}(tu|vw)'^{(2)}\hat{E}_{tu,vw} - \sqrt{\frac{\omega}{2}}\sum_{tu}(\mathbf{U}^T\mathbf{dU})_{tu}\hat{E}_{tu}(\hat{b}^\dagger + \hat{b}) + \sqrt{\frac{\omega}{2}}(\langle d_e\rangle - 2\sum_i(\mathbf{U}^T\mathbf{dU})_{ii})(\hat{b}^\dagger + \hat{b}) \tag{S28}$$

The stationary condition for CI coefficient is given by:

$$(\mathbf{H}_{CS-PF}^{CAS(2)} - E^{(2)}(T))\mathbf{C} = 0 \tag{S29}$$

A flowchart of a CASSCF macroiteration is shown in FigureS3. At the beginning of each macroiteration, the **J** and **K** integrals are updated using the rotation matrix **U** obtained from previous macroiteration. Subsequent CI optimization is performed until the residual norms of the target roots fall below a predefined convergence threshold. The convergence of the $E^{(2)}(\mathbf{T})$ model can be slow for rotations between inactive and active orbitals; therefore, additional optimization of such internal rotations is required. In internal optimization, the full energy expression E(**U**)

is optimized but the orbital gradient and Hessian contain only internal rotation parameters. Upon obtaining an updated $\mathbf{U}$ from internal optimization, the block $(kl|mn)$ of the $\mathbf{J}$ and $\mathbf{K}$ integrals are recalculated via a partial four-index transformation restricted to the occupied-occupied block of $\mathbf{U}$, followed by CI optimization until both the internal orbital optimization and CI optimization are converged. The partial four-index transformation in internal optimization scales as $\mathscr{O}(n_{occ}^5)$, which is negligible compared to the full four-index transformation (scales as $\mathscr{O}(n_{orb}^5)$). At the end of the internal optimization, the $\mathbf{J}$ and $\mathbf{K}$ integrals are partially transformed using the occupied-occupied block of $\mathbf{U}$. For example the $\mathbf{J}$ integral is transformed as:

$$J_{m'n'}^{k'l'} = \sum_{klmn} U_{mm'} U_{nn'} U_{kk'} U_{ll'} J_{mn}^{kl} \tag{S30}$$

$$J_{an'}^{k'l'} = \sum_{kln} U_{nn'} U_{kk'} U_{ll'} J_{an}^{kl} \tag{S31}$$

$$J_{ab}^{k'l'} = \sum_{kl} U_{kk'} U_{ll'} J_{ab}^{kl} \tag{S32}$$

$$J_{n'a}^{k'l'} = J_{an'}^{l'k'} \tag{S33}$$

The most computational demanding step (Eq. S32) scales no more than $\mathscr{O}(n_{orb}^2 n_{occ}^3)$. Therefore, the computational cost of this step is also inexpensive. After the internal optimization and microiteration optimization steps, further correction of internal rotations can be performed by repeating the internal transformation and microiteration optimization. The details can be found in Ref. 1,2.

Orbital optimization

Given an orbital gradient $\tilde{\mathbf{a}}$ and an orbital hessian $\tilde{\mathbf{H}}$, a second-order orbital optimization can be solved efficiently using the solution of the trust region subproblem:

$$\min f = \mathbf{x}^T \tilde{\mathbf{a}} + \frac{1}{2} \mathbf{x}^T \tilde{\mathbf{H}} \mathbf{x} \quad s.t. \quad ||\mathbf{x}|| \leq \Delta \tag{S34}$$

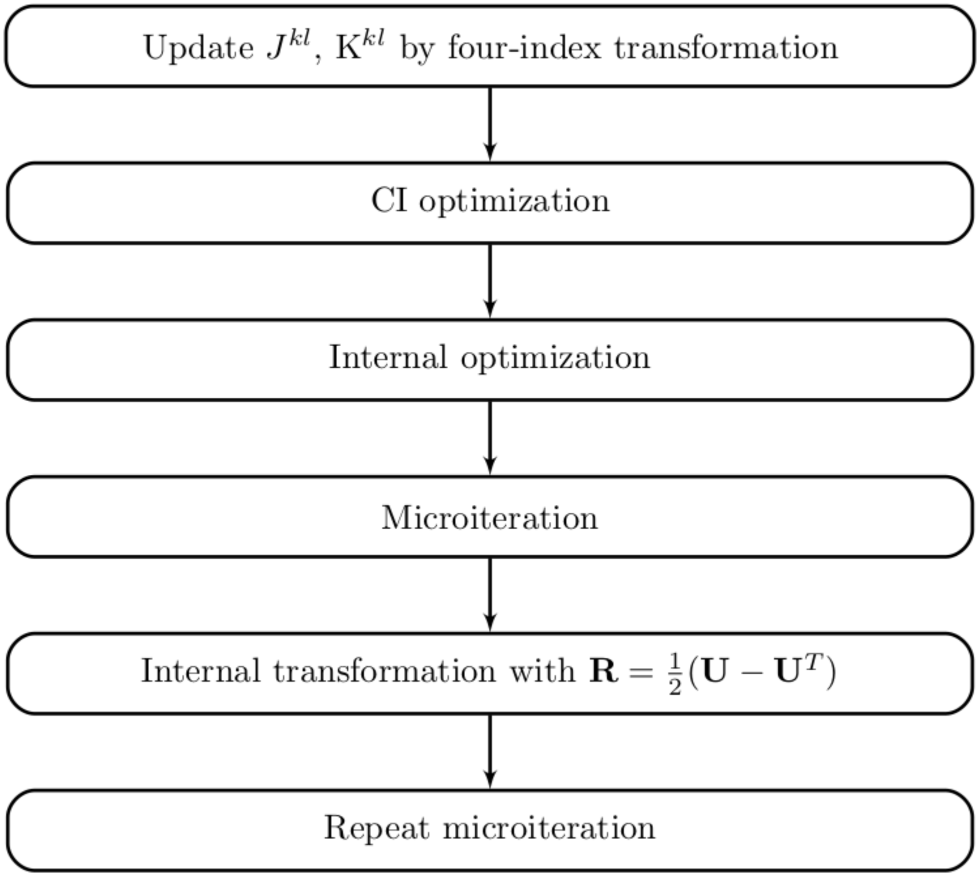

Figure S3: Flow diagram of a QED-CASSCF macroiteration.

Here, we minimize $f(\mathbf{x})$ as a quadratic model function of the variable $\mathbf{x}$, which represents the orbital step containing non-redundant elements of $\mathbf{R}$. The step $\mathbf{x}$ satisfies the boundary condition $||\mathbf{x}|| \leq \Delta$, where Δ is a predefined trust radius. While we use the $E^{(2)}(\mathbf{T})$ model to illustrate the application of the trust region subproblem, this method is also applicable to internal optimization procedure. The necessary and sufficient conditions to guarantee that a pair $(\lambda_*, \mathbf{x}_*)$ is the optimum solution of Eq. S34 are:[3]

$$\lambda_* \leq 0 \tag{S35}$$

$$||x_*|| \leq \Delta \tag{S36}$$

$$(\tilde{\mathbf{H}} - \lambda_* \mathbf{I})\mathbf{x}_* = \tilde{\mathbf{a}} \tag{S37}$$

$$\lambda_*(||\mathbf{x}_*|| - \Delta) = 0 \tag{S38}$$

$$(\tilde{\mathbf{H}} - \lambda_* \mathbf{I}) \succeq 0 \tag{S39}$$

The trust radius Δ can be dynamically adjusted using a modified Fletcher's algorithm[3] to ensure that the model function accurately approximate the objective function. The step length adjustment can be described as follows. We compare the model function and the objective function by considering the ratio:

$$r = \frac{\Delta E_{act}}{\Delta E_{pred}} \tag{S40}$$

$$\Delta E_{act} = (E^{(2)}(T))^k - (E^{(2)}(T))^{k-1} \tag{S41}$$

$$\Delta E_{pred} = \mathbf{x}^T \tilde{\mathbf{a}} + \frac{1}{2}\mathbf{x}^T \tilde{\mathbf{H}} \mathbf{x} \tag{S42}$$

where ΔE_{act} is the actual energy change of the $E^{(2)}(\mathbf{T})$ model after each update of the orbital rotation matrix $\mathbf{U}$ and ΔE_{pred} is the predicted energy change. Based on the value of r, we decide to accept or reject step and adjust the trust radius:

- If $r < 0$: The actual energy increases and we reject computed step, restart the current orbital optimization step with $\Delta_{k+1} = 0.5 * \Delta_k$

- If $0 \leq r \leq 0.25$: $\Delta_{k+1} = 0.7 * \Delta_k$
- If $r > 0.75$: $\Delta_{k+1} = \min(1.2 * \Delta_k, 0.75)$
- If $0.25 < r \leq 0.75$: $\Delta_{k+1} = \Delta_k$

In the quantum chemistry literature, the trust region subproblem is typically reformulated as a parametrized eigenvalue problem:

$$\mathbf{A}(\alpha)\mathbf{y}(\alpha) = \lambda(\alpha)\mathbf{y}(\alpha) \tag{S43}$$

where:

$$\mathbf{A}(\alpha) = \begin{pmatrix} 0 & \alpha\tilde{\mathbf{a}}^T \\ \alpha\tilde{\mathbf{a}} & \tilde{\mathbf{H}} \end{pmatrix}, \quad \mathbf{y}(\alpha) = \begin{pmatrix} 1 \\ \kappa(\alpha) \end{pmatrix} \tag{S44}$$

The second row of this equation can be rewritten as:

$$(\tilde{\mathbf{H}} - \lambda(\alpha)\mathbf{I})\alpha^{-1}\kappa(\alpha) = -\tilde{\mathbf{a}} \tag{S45}$$

The lowest eigenvalue (λ_1) of the parametrized eigenvalue problem S43 cannot exceed the smallest eigenvalue of $\tilde{\mathbf{H}}$ (μ_1) due to Cauchy's interlacing theorem. By following the lowest root of this eigenvalue problem, we can navigate toward regions where the Hessian becomes positive definite. The step length can be adjusted through appropriate selection of α. When $\lambda \leq 0$, $\mathbf{x} = \alpha^{-1}\kappa$ is the boundary solution of problem S34. This problem is typically solved using a modified Davidson algorithm. During each Davidson iteration, a search subspace is constructed, and the lowest Ritz pairs are computed with varying values of α until a step satisfying the boundary condition is identified. The search for the optimal value of α employs the bisection method.[4] Beginning with $\alpha = 1$, we incrementally increase α by a factor of 10 until determining an interval $[\alpha_{min}, \alpha_{max}]$ where $\mathbf{x}_{min} = \alpha_{min}^{-1}\kappa \leq \Delta$ and $\mathbf{x}_{max} = \alpha_{max}^{-1}\kappa \geq \Delta$. Subsequently, bisection search proceeds within this interval. However, this approach presents two limitations. First, there is no guarantee that the

optimum value of α exists within the interval $[1,\infty)$. Second, if the gradient $\tilde{\mathbf{a}}$ is orthogonal to the lowest eigenspace $\mathbf{S}_1$ of $\tilde{\mathbf{H}}$, there may not exist an α value corresponding to $\mathbf{x} = \alpha^{-1}\kappa = \Delta$ depends on the value of trust radius. This scenario is known as the "hard case" in trust region subproblem literature. To illustrate this problem, we analyze the function $||s(\lambda)|| = ||(\tilde{\mathbf{H}} - \lambda\mathbf{I})^{-1}\tilde{\mathbf{a}}||$ for a hypothetical Hessian with three eigenvalues -1 , -0.5 and 2 (Figure S4). In this example, the gradient is orthogonal to the first eigenvector of the Hessian, thus the function $s(\lambda)$ does not exhibit a pole at the first eigenvalue. The optimum λ_* should be determined within the range $(-\infty,-1]$, however, there exists a limiting value for the possible trust radius in this interval. This critical value $\tilde{\Delta} = 2.03$ corresponds to the intersection of the vertical dash line passing through the lowest root and the function $\|s(\lambda)\|$ within this interval. A trust radius exceeding this value can not satisfy the equation $||s(\lambda)|| = \Delta$, as demonstrated for $\Delta = 4$. These challenges frequently emerge in our practical applications. To achieve improved step length control, we adapt the LSTRS method described in Ref. 5,6, incorporating several modifications to the convergence criteria. In this method, we solve the eigenvalue problem:

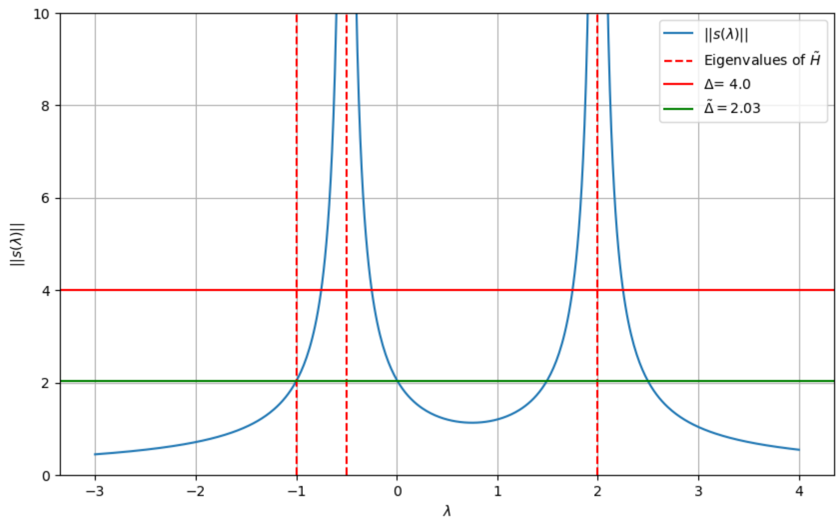

Figure S4: Illustration of the hard case when the function $\|s(\lambda)\|$ has no pole at the first eigenvalue of the Hessian.

$$\mathbf{B}(\alpha)\mathbf{y}(\alpha) = \lambda(\alpha)\mathbf{y}(\alpha) \tag{S46}$$

where:

$$\mathbf{B}(\alpha) = \begin{pmatrix} \alpha & \tilde{\mathbf{a}}^T \\ \tilde{\mathbf{a}} & \tilde{\mathbf{H}} \end{pmatrix}, \quad \mathbf{y}(\alpha) = \begin{pmatrix} 1 \\ \mathbf{x}(\alpha) \end{pmatrix} \tag{S47}$$

The benefit of this method is that it searches for α in an interval $[\alpha_L, \alpha_U]$ that is guaranteed by theory to contain the optimum value of α. In the "easy case", for any values α_k in the kth iteration, the eigenvector of the lowest root of $B(\alpha)$ can always be safely normalized to have the form $(1, \mathbf{x_k}^T)^T$. The LSTRS method then uses the pair $\{\lambda_k, \mathbf{x_k}\}$ with the current α_k in a one-point or two-point interpolating scheme to evaluate α_{k+1}. The one-point interpolating scheme is applied in the iteration $k = 0$ to provide a new update of α as follows:

$$\alpha_1 = \alpha_0 + \frac{\alpha_0 - \lambda_0}{||\mathbf{x}_0||}\left(\frac{\Delta - ||\mathbf{x}_0||}{\Delta}\right)\left(\Delta + \frac{1}{||\mathbf{x}_0||}\right) \tag{S48}$$

The two-point interpolating scheme is applied in the iteration $k \geq 1$ using information from the kth and $(k-1)^{th}$ iterations:

$$\hat{\lambda} = \frac{\lambda_{k-1}||\mathbf{x}_{k-1}||(||\mathbf{x}_k|| - \Delta) + \lambda_k||\mathbf{x}_k||(\Delta - ||\mathbf{x}_{k-1}||)}{\Delta(||\mathbf{x}_k|| - ||\mathbf{x}_{k-1}||)} \tag{S49}$$

$$\Omega = \frac{\lambda_k - \hat{\lambda}}{\lambda_k - \lambda_{k-1}} \tag{S50}$$

$$\begin{aligned} \alpha_{k+1} &= \Omega\alpha_{k-1} + (1-\Omega)\alpha_k \\ &+ \frac{||\mathbf{x}_{k-1}||\,||\mathbf{x}_k||(||\mathbf{x}_k|| - ||\mathbf{x}_{k-1}||)}{\Omega||\mathbf{x}_k|| + (1-\Omega)||\mathbf{x}_{k-1}||}\frac{(\lambda_{k-1} - \hat{\lambda})(\lambda_k - \hat{\lambda})}{\lambda_k - \lambda_{k-1}} \end{aligned} \tag{S51}$$

In the hard case when $\tilde{\mathbf{a}} \perp \mathbf{S}_1$, for all α values greater than a critical value $\tilde{\alpha}$, the eigenvector corresponding to λ_1 has the form $(0, \mathbf{q}^T)^T$ where $\mathbf{q} \in \mathbf{S}_1$. Therefore, the previous interpolating schemes can not be applied for this eigenvector. However, for all α values, there always exists an

eigenvector that can be safely normalized to have the first component one. The LSTRS method uses another eigenvector corresponding to the second (or close to the second) eigenvalue that can be normalized to obtain a new interpolation value of alpha. The solution for the hard case is obtained when the critical value $\tilde{\alpha}$ is found. For this value, the matrix $\mathbf{B}(\tilde{\alpha})$ has at least two eigenvectors, $(0, \mathbf{q}^T)^T$ and $(1, \mathbf{p}^T)^T$, which correspond to $\lambda_1 = \mu_1$. The solution to the hard case is then given by:

$$\mathbf{x} = \mathbf{p} + b\mathbf{q} \tag{S52}$$

The scalar b can be chosen to obtain the boundary solution. The equation $||\mathbf{p} + b\mathbf{q}|| = \Delta$ has two solutions; between these two values, the value of b with a smaller magnitude is the optimum choice proved by theory.

The LSTRS algorithm is described in Figure S5. For a unitary eigenvector $(\nu_n, \mathbf{u}_n^T)^T$ corresponding to the nth root of $\mathbf{B}(\alpha_k)$, the value ν_n is considered to be "small" and potential hard case has been detected if $\|\tilde{\mathbf{a}}\||\nu_n| \leq \varepsilon_\nu \sqrt{1-\nu_n^2}$ for some $\varepsilon_\nu \in (0,1)$. When both $(\nu_1, \mathbf{u}_1^T)^T\}$ and $(\nu_2, \mathbf{u}_2^T)^T$ exhibit concurrent small first components, α-adjustment and root recomputation become necessary. As we update the optimal range $[\alpha_L, \alpha_U]$, we must implement a safeguard strategy at the end of each iteration to ensure that the new α value remains within the current interval $[\alpha_L, \alpha_U]$. The following conditions are checked at each iteration to stop the algorithm:

- If $\left| \left\| \frac{\mathbf{u}_1}{\nu_1} \right\| - \Delta \right| \leq \varepsilon_\Delta * \Delta$ and $\lambda_1 \leq 0$ for a given $\varepsilon_\Delta \in (0,1)$: The boundary solution has been found. The step is given by $\mathbf{x}_* = \mathbf{u}_1/\nu_1$.

- If $\|\mathbf{u_1}\| < \Delta|\nu_1|$ and $\lambda_1 > -\varepsilon_{Int}$ for a given $\varepsilon_{Int} =\in [0,1)$: The optimum solution is the Newton step $\lambda_* = 0$ and $\mathbf{x}_* = -\tilde{\mathbf{H}}^{-1}\tilde{\mathbf{a}}$. The LSTRS iteration is terminated and we use MINRES from SCIPY package to calculate the Newton step.

- If we can find a vector $\widetilde{\mathbf{x}}$ such that $\|\widetilde{\mathbf{x}}\| = \Delta$ and:

$$f(\mathbf{x}_*) \leq f(\widetilde{\mathbf{x}}) \leq (1 - \varepsilon_{HC}) f(\mathbf{x}_*) \tag{S53}$$

  for a given $\varepsilon_{HC} \in (0,1)$, then $f(\widetilde{\mathbf{x}})$ is sufficiently close to $f(\mathbf{x}_*)$ and $\widetilde{\mathbf{x}}$ is a quasi-optimal solution of the trust region subproblem. The conditions used to determine such a solution can be found in Figure S10. Finding a quasi-optimal solution will allow us to terminate the LSTRS iteration in the hard case or in the near-hard case (when the gradient is nearly orthogonal to the first eigenvector of $\tilde{\mathbf{H}}$).

- If the safeguarding interval is too small: If $|\alpha_U - \alpha_L| \leq \varepsilon_\alpha \max\{|\alpha_U|, |\alpha_L|\}$ for $\varepsilon_\alpha \in (0,1)$, the iteration is stopped. In the exact hard case, we typically identify the critical value $\tilde{\alpha}$ described previously and the two eigenvectors $(0, \mathbf{q}^T)^T$ and $(1, \mathbf{p}^T)^T$. The boundary solution can be subsequently calculated using the Eq. S52. In the near-hard case, the function $\|s(\lambda)\|$ maintains a pole at $\lambda = \mu_1$. However, the value $\lambda_* - \mu_1$ may be prohibitively small, rendering it impossible to determine the boundary solution or quasi-optimum solution within the current tolerance threshold. In that case, if $\left\|\frac{\mathbf{u_1}}{v_1}\right\| < \Delta$ we accept a step $\mathbf{x} = \frac{\mathbf{u}_1}{v_1}$. In contrast, if $\left\|\frac{\mathbf{u_1}}{v_1}\right\| > \Delta$, we scale the coordinates of the vector $\mathbf{u}_1$ to get a step $x = sgn(v_1)\Delta * \frac{\mathbf{u}_1}{\|\mathbf{u}_1\|}$.

The current scheme for solving large-scale trust region subproblems exhibits two key limitations. First, the parameter adjustment for α is decoupled from the eigenvalue root-finding process, overlooking potential convergence synergies between α and the evolving matrix $\mathbf{B}(\alpha)$. An iterative eigenvalue solver leveraging historical subspace information could enhance efficiency. Second, the mandatory computation of two roots introduces redundancy, as the second root is only required if the first root fails normalization (i.e., its first component deviates from unity). In Ref. 7, these problems have been addressed by using iterative projections methods. Using either nonlinear Arnoldi or Jacobi-Davidson method, one can reuse the search subspace from $\mathbf{B}(\alpha_k)$ to initialize subsequent iterations for $\mathbf{B}(\alpha_{k+1})$ and compute only the lowest root per iteration, selectively expanding the subspace when seeking a second root becomes necessary. In our implementation, we mitigate the

Algorithm 1 LSTRS

Input: $\tilde{H} \in \mathbb{R}^{n \times n}$, $\tilde{a} \in \mathbb{R}^n$, $\Delta > 0$, ε_Δ, ε_ν, ε_{HC}, $\varepsilon_\alpha \in (0,1)$, $\varepsilon_{Int} \in [0,1)$.
Output: λ^*, x^* satisfying conditions of the boundary solution.

1. Initialization
  1.1 Compute $\delta_U = \min(\tilde{H}_{ii} | i = 1, \ldots n)$, initialize $\alpha_U = \delta_U + ||\tilde{a}||\Delta$, set $\alpha_0 = \alpha_U$.
  1.2 Compute eigenpairs $\{\lambda_1(\alpha_0), (\nu_1, \mathbf{u}_1^T)^T\}$, $\{\lambda_i(\alpha_0), (\nu_2, \mathbf{u}_2^T)^T\}$ corresponding to smallest eigenvalue and the second (or close to the second) smallest eigenvalue of B_{α_0}.
  1.3 Initialize $\alpha_L = \lambda_1(\alpha_0) - \frac{||\tilde{a}||}{\Delta}$. Update $\delta_U = \min\left\{\delta_U, \lambda_1(\alpha_0) - \nu_1 * \frac{\tilde{\mathbf{a}}^T \mathbf{u}_1}{\mathbf{u}_1^T \mathbf{u}_1}\right\}$.
  1.4 Set $k = 0$.
2. Repeat
  2.1 Adjust α_k (Figure S8).
  2.2 If $\|\tilde{\mathbf{a}}\||\nu_1| > \varepsilon_\nu \sqrt{1 - \nu_1^2}$ then:
    Set $\lambda_k = \lambda_1(\alpha_k)$ and $x_k = \frac{u_1}{\nu_1}$.
    If $\|x_k\| < \Delta$ then $\alpha_L = \alpha_k$.
    If $\|x_k\| > \Delta$ then $\alpha_U = \alpha_k$.
   Else:
    Set $\lambda_k = \lambda_i(\alpha_k)$, $x_k = \frac{u_2}{\nu_2}$, and $\alpha_U = \alpha_k$.
   End if.
  2.3 Check for quasi-optimum solutions (Figure S10).
  2.4 If $k = 0$ then
    Compute α_{k+1} using (S48).
   Else
    If $(\Omega\|x_k\| + (1 - \Omega)\|x_{k-1}\|)(\lambda_k - \lambda_{k-1}) \neq 0$: Compute α_{k+1} using (S51).
    Else: $a_{k+1} = (a_L + a_U)/2$
    End if.
   End if.
  2.5 Safeguard α_{k+1} (Figure S9).
  2.6 Set $k = k + 1$.
Until convergence.

Figure S5: Outline of the LSTRS Algorithm.

first limitation by adopting the the P-space Davidson method described in Ref. 8 and seek two roots in every iteration. The ideas of this method can be summarized as follows. For any n_P orthonormal column vectors $P_1, P_2, ...P_{n_P}$ of length m where $m = n+1$ is the dimension of $\mathbf{B}$ ($n_P < m$), it is possible to find n_Q complementary orthonormal vectors $Q_1, Q_2, ...Q_{n_Q}$ ($n_P + n_Q = m$) such that $O = [P_1, P_2..., P_{n_P}, Q_1, Q_2, ...Q_{n_Q}]$ is an orthogonal matrix. The transformed matrix:

$$\bar{\mathbf{B}} = \mathbf{O}^T\mathbf{B}\mathbf{O} = \begin{pmatrix} \mathbf{P}^T\mathbf{B}\mathbf{P} & \mathbf{P}^T\mathbf{B}\mathbf{Q} \\ \mathbf{Q}^T\mathbf{B}\mathbf{P} & \mathbf{Q}^T\mathbf{B}\mathbf{Q} \end{pmatrix} \tag{S54}$$

preserves the eigenvalues of $\mathbf{B}$. In the P-space Davidson method, the initial search subspace $\mathbf{R}_0$ contains n_P vectors in the P-space, and in iteration k, the subspace $\mathbf{R}_k$ contains additional l vectors in the Q-space ($k \leq l \leq n_Q$) such that:

$$\bar{\mathbf{B}}_k = \begin{pmatrix} \mathbf{P}^T\mathbf{B}\mathbf{P} & \mathbf{P}^T\mathbf{B}\mathbf{Q}_k \\ \mathbf{Q}_k^T\mathbf{B}\mathbf{P} & \mathbf{Q}_k^T\mathbf{B}\mathbf{Q}_k \end{pmatrix} \tag{S55}$$

where $\bar{\mathbf{B}}_k$ is the projection of B onto the subspace $\mathbf{R}_k$. Approximate eigenvalues of the matrix $\mathbf{B}$ are obtained from:

$$\bar{\mathbf{B}}_k y_k = \lambda_k y_k \tag{S56}$$

The Davidson correction vector for the ith root is then given by:

$$r_j^i = \frac{[\mathbf{B}\mathbf{R}_k y_k^i - \lambda^i \mathbf{R}_k y_k^i]_j}{(\lambda_i - B_{jj})} \tag{S57}$$

which is added to the current search subspace after a Gramm-Schmidt orthonormalization. In the P-space Davidson method, the projection $\mathbf{P}^T\mathbf{B}\mathbf{P}$ is a principal submatrix of the matrix $\mathbf{B}$. The matrix P is a partial permutation matrix whose columns are unit vectors. Let $I = \{i_1, i_2...i_{n_P}\}$ be the set of indices corresponding to the non-zero rows of the matrix P. The projection matrix $\mathbf{P}^T\mathbf{B}\mathbf{P}$

can be obtained by selecting rows and columns of the matrix **B** indexed by the set I:

$$\mathbf{P}^T\mathbf{B}\mathbf{P} = B[I,I] \tag{S58}$$

Moreover, any vector $v \in \mathbb{R}^m$ can be projected onto P-space as:

$$Pv = v[I] \tag{S59}$$

Thus, there is no need to compute matrix-vector product of the matrix **B** with basic vectors in the P-space. To obtain the initial subspace P, we sort the array containing $\frac{|\tilde{a}_{rk}|}{\tilde{H}_{rk,rk}}$ in ascending order and form a set D from the first $(n_P - 1)$ indices that sorts the array. The set D captures the indices of the most important orbital rotations. Since the projected matrix should preserve the structure of **B** by including the element $B_{00} = \alpha$, the basis vectors for P is constructed from the set $I = \{D, 0\}$. In our work, the maximum value for n_p is set to be 200.

In practice, we only solve the trust region subproblem in the global region of the optimization. In the local region where the gradient norm is small enough ($||\tilde{\mathbf{a}}|| \leq \varepsilon_c$), it is assumed that the Hessian is positive definite and we switch to Newton method. The step is then calculated using MINRES from SCIPY.

Convergence Criteria

As described earlier, we implement the "Uncoupled CI" algorithm from Reference 8. This approach minimizes the number of CI optimizations during each microiteration by increasing orbital optimization frequency in early macroiterations when the orbital gradient norm is large. Subsequently, as the orbital gradient norm decreases in later macroiterations, the algorithm typically performs one orbital optimization and one CI optimization per microiteration (see Figure S6). The microiteration convergence criterion is presented in Figure S7. For internal optimization convergence, two conditions must be satisfied: the orbital gradient norm is smaller than 1.0×10^{-4} a.u., and the CI residual vector norm is smaller than 1.0×10^{-6} a.u.. The initial trust radius is 0.4 a.u..

One orbital optimization step $\rightarrow$ step $\mathbf{x}$
if $\|\mathbf{x}\| > 0.1$ **then**
  $N_{\text{micro}} = 5$
  $N_{\text{orb}} = 4$
else if $\|\mathbf{x}\| > 0.01$ **then**
  $N_{\text{micro}} = 7$
  $N_{\text{orb}} = 3$
else
  $N_{\text{micro}} = 20$
  $N_{\text{orb}} = 1$
end if
$N_{\text{orb}} = 1$ orbital optimization steps
do microiterations $= 1, N_{\text{micro}} - 1$
  CI Davidson optimization (5 iterations)
  N_{orb} orbital optimization steps
  if convergence $\rightarrow$ exit loop
end do

Figure S6: Uncoupled CI strategy

$\varepsilon_{acc} = \min(0.01\|\tilde{\mathbf{a}}^0\|, \|\tilde{\mathbf{a}}^0\|^2)$
$i =$ microiteration
if total gradient $\sqrt{\|\tilde{\mathbf{a}}^i\|^2 + \|\mathbf{r}_{ci}\|^2} < \varepsilon_{acc}$
  or
  $\|\tilde{\mathbf{a}}^i\| < \max(0.1\varepsilon_{acc}, 1.0 \times 10^{-7})$
  or
  $|E^i - E^{i-1}| < \max(0.01\varepsilon_{acc}, 1.0 \times 10^{-10})$ and $i \geq 3$ **then**
  convergence reached
end if

Figure S7: Microiteration convergence criterion

Step 2.1. Adjust α_k

Input: $\delta_U, \varepsilon_\nu, \varepsilon_\alpha \in (0,1)$, α_L, α_U, α_k with $\alpha_k \in [\alpha_L, \alpha_U]$.
Output: δ_U, α_k, $\{\lambda_1(\alpha_k), (\nu_1, \mathbf{u}_1^T)^T\}$ and $\{\lambda_i(\alpha_k), (\nu_2, \mathbf{u}_2^T)^T\}$.

Set $\alpha = \alpha_k$
if $k > 0$ **then**
  Compute eigenpairs $\{\lambda_1(\alpha), (\nu_1, \mathbf{u}_1^T)^T\}$ and $\{\lambda_i(\alpha), (\nu_2, \mathbf{u}_2^T)^T\}$, corresponding to the smallest eigenvalue and the second (or close to the second smallest) eigenvalue of B_α.
  Update $\delta_U = \min\left\{\delta_U, \lambda_1 - \nu_1 * \frac{\tilde{\mathbf{a}}^T \mathbf{u}_1}{\mathbf{u}_1^T \mathbf{u}_1}\right\}$.
end if
while $\|\tilde{\mathbf{a}}\||\nu_1| \leq \varepsilon_\nu \sqrt{1-\nu_1^2}$ and $\|\tilde{\mathbf{a}}\||\nu_2| \leq \varepsilon_\nu \sqrt{1-\nu_2^2}$ and $|\alpha_U - \alpha_L| > \varepsilon_\alpha * \max\{|\alpha_L|, |\alpha_U|\}$ **do**
  $\alpha_U = \alpha$
  $\alpha = (\alpha_L + \alpha_U)/2$
  Compute $\{\lambda_1(\alpha), (\nu_1, \mathbf{u}_1^T)^T\}$ and $\{\lambda_i(\alpha), (\nu_2, \mathbf{u}_2^T)^T\}$.
  Update $\delta_U = \min\left\{\delta_U, \lambda_1 - \nu_1 * \frac{\tilde{\mathbf{a}}^T \mathbf{u}_1}{\mathbf{u}_1^T \mathbf{u}_1}\right\}$.
end while
Set $\alpha_k = \alpha$

Figure S8: Adjust α_k

Step 2.5 Safeguard α_{k+1}

Input: α_{k+1} computed by step 2.4 of Algorithm 1, $\delta_U \geq \mu_1$, α_L, α_U, $\phi_i = -\tilde{\mathbf{a}}^T \mathbf{x}_i$, and $\phi_i' = \|\mathbf{x}_i\|^2$, for $i = k-1, k$.
Output: Safeguarded value for α_{k+1}.

if $\alpha_{k+1} \notin [\alpha_L, \alpha_U]$ **then**
  if $k = 0$ **then**
    $\alpha_{k+1} = \delta_U + \phi_k + \phi_k'(\delta_U - \lambda_k)$
  else if $\|x_k\| < \|x_{k-1}\|$ **then**
    $\alpha_{k+1} = \delta_U + \phi_k + \phi_k'(\delta_U - \lambda_k)$
  else
    $\alpha_{k+1} = \delta_U + \phi_{k-1} + \phi_{k-1}'(\delta_U - \lambda_{k-1})$
  end if
  if $\alpha_{k+1} \notin [\alpha_L, \alpha_U]$ **then**
    Set $\alpha_{k+1} = (\alpha_L + \alpha_U)/2$
  end if
end if

Figure S9: Safeguard α_{k+1}

Step 2.3 Check for quasi-optimal solution

Input: λ_1, $(v_1, \mathbf{u}_1^T)^T$, λ_i, $(v_i, \mathbf{u}_i^T)^T$, $\varepsilon_{HC} \in (0,1)$.

Output: True or False: return True if quasi-optimal solution has been found and also return the solution $\widetilde{\lambda}$, $\widetilde{\mathbf{x}}$ otherwise return False.

$\eta = \frac{\varepsilon_{HC}}{1-\varepsilon_{HC}}$

if $(1+\Delta)(v_1^2+v_i^2) > 1$ **then**

$$\tau_1 = \frac{v_1 - v_i\sqrt{(1+\Delta^2)(v_1^2+v_i^2)-1}}{(v_1^2+v_i^2)\sqrt{1+\Delta^2}}, \quad \tau_2 = \frac{v_i + v_1\sqrt{(1+\Delta^2)(v_1^2+v_i^2)-1}}{(v_1^2+v_i^2)\sqrt{1+\Delta^2}}$$

else if $(1+\Delta)(v_1^2+v_i^2) = 1$ **then**

$$\tau_1 = \frac{v_1}{\sqrt{v_1^2+v_i^2}}, \quad \tau_2 = \frac{v_i}{\sqrt{v_1^2+v_i^2}}$$

end if

$$\widetilde{\mathbf{x}} = \frac{\tau_1\mathbf{u}_1 + \tau_2\mathbf{u}_i}{\tau_1 v_1 + \tau_2 v_i}, \quad \widetilde{\lambda} = \tau_1^2\lambda_1 + \tau_2^2\lambda_i, \quad f(\widetilde{\mathbf{x}}) = \widetilde{\mathbf{x}}^T\tilde{\mathbf{a}} + \frac{1}{2}\widetilde{\mathbf{x}}^T\tilde{\mathbf{H}}\widetilde{\mathbf{x}}$$

if $(\lambda_i - \lambda_1)\tau_2^2(1+\Delta^2) \leq -2\eta f(\widetilde{\mathbf{x}})$ **then**

  $\widetilde{\lambda}, \widetilde{x}$ is the quasi-optimal pair.

else

  if $(1+\Delta)(v_1^2+v_i^2) > 1$ **then**

$$\tau_1 = \frac{v_1 + v_i\sqrt{(1+\Delta^2)(v_1^2+v_i^2)-1}}{(v_1^2+v_i^2)\sqrt{1+\Delta^2}}, \quad \tau_2 = \frac{v_i - v_1\sqrt{(1+\Delta^2)(v_1^2+v_i^2)-1}}{(v_1^2+v_i^2)\sqrt{1+\Delta^2}}$$

$$\widetilde{\mathbf{x}} = \frac{\tau_1\mathbf{u}_1 + \tau_2\mathbf{u}_i}{\tau_1 v_1 + \tau_2 v_i}, \quad \widetilde{\lambda} = \tau_1^2\lambda_1 + \tau_2^2\lambda_i, \quad f(\widetilde{\mathbf{x}}) = \widetilde{\mathbf{x}}^T\tilde{\mathbf{a}} + \frac{1}{2}\widetilde{\mathbf{x}}^T\tilde{\mathbf{H}}\widetilde{\mathbf{x}}$$

    if $(\lambda_1 - \lambda_i)\tau_2^2(1+\Delta^2) \leq -2\eta f(\widetilde{\mathbf{x}})$ **then**

      $\widetilde{\lambda}, \widetilde{x}$ is the quasi-optimal pair.

    end if

  end if

end if

Figure S10: Quasi-optimum condition

The values used for different convergence criteria are: $\varepsilon_{\Delta} = 1.0 \times 10^{-3}$, $\varepsilon_{Int} = 1.0 \times 10^{-8}$, $\varepsilon_{HC} = 1.0 \times 10^{-6}$, $\varepsilon_{\alpha} = 1.0 \times 10^{-8}$, $\varepsilon_{v} = 1.0 \times 10^{-4}$, $\varepsilon_{c} = 1.0 \times 10^{-3}$ all in a.u. In the calculations described in the main text, we consider the energy to be converged when the energy difference between successive macroiterations falls below 1.0×10^{-9} E_{h}. For the origin invariance study, we employed a stricter convergence criterion of $|E^{k}_{macro} - E^{k-1}_{macro}| \leq 1.0 \times 10^{-12}$ E_{h}.

References

(1) Kreplin, D. Multiconfiguration self-consistent field methods for large molecules. Ph.D. thesis, 2020.

(2) Werner, H.; Knowles, P. J. A second order multiconfiguration SCF procedure with optimum convergence. *The Journal of Chemical Physics* **1985**, *82*, 5053–5063.

(3) Nocedal, J.; Wright, S. *Numerical Optimization*; Springer New York: New York, NY, 2006; pp 66–100.

(4) Helmich-Paris, B. A trust-region augmented Hessian implementation for restricted and unrestricted Hartree–Fock and Kohn–Sham methods. *The Journal of Chemical Physics* **2021**, *154*, 164104.

(5) Rojas, M.; Santos, S. A.; Sorensen, D. C. A New Matrix-Free Algorithm for the Large-Scale Trust-Region Subproblem. *SIAM Journal on Optimization* **2001**, *11*, 611–646.

(6) Rojas, M.; Santos, S. A.; Sorensen, D. C. Algorithm 873: LSTRS: MATLAB software for large-scale trust-region subproblems and regularization. *ACM Trans. Math. Softw.* **2008**, *34*.

(7) Lampe, J.; Rojas, M.; Sorensen, D. C.; Voss, H. Accelerating the LSTRS Algorithm. *SIAM Journal on Scientific Computing* **2011**, *33*, 175–194.

(8) Kreplin, D. A.; Knowles, P. J.; Werner, H.-J. Second-order MCSCF optimization revisited. I. Improved algorithms for fast and robust second-order CASSCF convergence. *The Journal of Chemical Physics* **2019**, *150*, 194106.
